# Regional Scale Prioritisation for Key Ecosystem Services, Renewable Energy Production and Urban Development

**DOI:** 10.1371/journal.pone.0107822

**Published:** 2014-09-24

**Authors:** Stefano Casalegno, Jonathan J. Bennie, Richard Inger, Kevin J. Gaston

**Affiliations:** Environment & Sustainability Institute, University of Exeter, Penryn, Cornwall, United Kingdom; Federal University of Goiás, Brazil

## Abstract

Although the importance of addressing ecosystem service benefits in regional land use planning and decision-making is evident, substantial practical challenges remain. In particular, methods to identify priority areas for the provision of key ecosystem services and other environmental services (benefits from the environment not directly linked to the function of ecosystems) need to be developed. Priority areas are locations which provide disproportionally high benefits from one or more service. Here we map a set of ecosystem and environmental services and delineate priority areas according to different scenarios. Each scenario is produced by a set of weightings allocated to different services and corresponds to different landscape management strategies which decision makers could undertake. Using the county of Cornwall, U.K., as a case study, we processed gridded maps of key ecosystem services and environmental services, including renewable energy production and urban development. We explored their spatial distribution patterns and their spatial covariance and spatial stationarity within the region. Finally we applied a complementarity-based priority ranking algorithm (zonation) using different weighting schemes. Our conclusions are that (i) there are two main patterns of service distribution in this region, clustered services (including agriculture, carbon stocks, urban development and plant production) and dispersed services (including cultural services, energy production and floods mitigation); (ii) more than half of the services are spatially correlated and there is high non-stationarity in the spatial covariance between services; and (iii) it is important to consider both ecosystem services and other environmental services in identifying priority areas. Different weighting schemes provoke drastic changes in the delineation of priority areas and therefore decision making processes need to carefully consider the relative values attributed to different services.

## Introduction

The importance of incorporating consideration of ecosystem service benefits into land use planning and decision making for sustainable development has been much highlighted [1]–[7]. The challenges, however, remain substantial (Table 1). First, in the main, analyses of the distributions of ecosystem services have not been conducted at the spatial extents and resolutions that are likely to be most relevant to political and planning processes [8], [9]. They have typically been mapped over broader extents and coarser resolutions (which are nonetheless often important for strategic reasons). Second, there is almost invariably a paucity of original data on the distribution of ecosystem services in a given region that prevents them from being adequately directly mapped, and forces heavy reliance on methods of benefits transfer (i.e. using data from studies conducted in other regions) [10]–[12]. Third, this often results in analyses being focused on those few services for which information is available (commonly the same core ones in many studies), rather than on those which may regionally be most relevant [8], [13]. Fourth, to date there has been limited application of analytical tools that are well suited to addressing the issues that are posed by the often spatially complex patterns of variation and covariation in ecosystem services. In particular, methods have seldom been used that address complementarity and that enable the priority ranking of areas rather than simply the identification of sets of areas that provide some minimal cost solution [13]–[17]. Finally, where spatial planning issues have been considered, these have focused almost exclusively on ecosystem services and biodiversity, and/or possible trade-offs with urban development [13], . These analyses have however neglected what we might call ‘environmental services’, particularly those benefits from the environment not readily directly attributed to ecosystems per se (e.g. wind and solar energy, space for human living); here we treat ecosystem services as a subset of environmental services.

**Table 1:** Appraisal of how a sample of studies have accounted for the challenges of incorporating ecosystem service benefits into land use planning and decision making (see Introduction).

	Appropriate	Appropriate	Avoid	Includes key	Use spatial	Includes other
	extent	resolution	benefit	ecosystem	analytical	environmental
			transfer	services	tools	services
Bateman et al. 2013 [9]	**	-	**	-	**	-
Estoque et al (2013) [74]	*	**	-	-	-	-
Bagstad (2012) [75]	***	*	-	-	**	*
Viglizzo et al (2012) [76]	***	***	*	**	*	-
Frank et al (2012) [8]	-	**	-	-	**	-
Goldstein et al (2012) [77]	**	**	-	-	**	*
Koschke et al. (2012) [78]	**	**	-	*	**	-
Scolozzi et al (2012) [79]	*	*	-	*	-	-
Trepel et al. (2012) [80]	*	*	*	*	*	-
Chan et al (2011) [81]	**	**	**	*	**	-
Moilanen (2011) [16]	*	*	**	*	**	-
Nelson et al. (2009) [13]	*	*	**	-	**	*
Chan et al (2006) [14]	**	**	*	*	**	-
Zhao et al. (2004) [82]	***	**	-	*	-	-

**fulfilled;

*partially fulfilled;

- not fulfilled.

These understandable constraint have limited knowledge of some fundamental issues, including how environmental services are distributed across regions, the patterns of covariance and co-occurrence in these distributions, and the distribution of priority areas for environmental service provision. Here we aim to address these issues in considering the spatial prioritisation of environmental services for the county of Cornwall, U.K. Cornwall is particularly suitable for such a study, given that (i) it is a relatively discrete geopolitical unit, it is a peninsula extending over ∼3.500 km^2^ that is bounded by sea for the majority of its border; (ii) data on the spatial distribution of environmental services is available at a resolution (1 km^2^) relevant to decision makers in their planning processes (although finer resolutions would typically be required to match with scales of individual developments), and for which it is possible to mostly avoid undue reliance on benefit transfer functions; and (iii) key regional strategic, policy and funding bodies have aspirations for the region to be a leader in understanding the value of its environmental resources and in reducing the pressure placed on these (e.g. Cornwall and Isles of Scilly Local Enterprise Partnership 2012).

Of course, it is difficult, and perhaps impossible, objectively to identify which ecosystem services are of the greatest importance for any given region. However, it is possible to recognise some of particular significance. The value of the services we are discussing is not always monetary (as for urban development) but is related to the long-term sustainability of the region which may contrast with short term monetary value. Across the four main categories of ecosystem services (cultural, provisioning, regulating, supporting) defined by the Millennium Ecosystem Assessment [19], we selected several as being of disproportionate significance for Cornwall: tourism, recreation and aesthetic services within cultural services; agriculture as a provisioning service; above and below ground carbon stocks and flood mitigation as regulating services; and plant production as a supporting service. Tourism and recreation are the largest economic (£896 million in 2006, equal to 13% of total gross value added) and employment sectors in Cornwall (14% of total employees) [20], with the aesthetic value of ecosystems (particularly in the coastal zones) being a leading draw to visitors. Agriculture is also a major industry, comprising the most extensive land use in the county (∼84%; estimated from the European Environmental Agency [21]) and 2.3% of the economy, and accounting for 7.5% of all employees [20]. Although not extensive, areas of Cornwall approach the peak densities of carbon storage for England [22], and protection of these zones is a high priority. Flooding has been a persistent concern in the region [23], having caused significant damage to property and businesses in recent years, and thus flood mitigation is a valued ecosystem service. As a supporting service, we selected plant production as an ecosystem function necessary for multiple other ecosystem services. For example, plant production is linked to agricultural production and other provisioning services (such as forestry); it is linked to water-cycle related ecosystem services such as water quality and flood mitigation; it is linked to short-term carbon sequestration; and it may provide habitat for a broad spectrum of crop-pest natural enemies [24] again linked to forestry and agricultural production. Plant production and aboveground carbon stocks may have similar distribution patterns in homogeneous landscapes. In fragmented landscapes such as Cornwall, where crop rotation, pasture, woodlands and natural vegetation occur, production and aerial biomass can differ substantially.

Besides ecosystem services, it is also possible to identify some key environmental services within Cornwall that are not directly attributable to ecosystems, although they may be substantially influenced by them. The region is seen as being potentially significant for wind and particularly solar renewable energy production, and this is a fast growing sector [25]. Cornwall also has a population of ∼532,000 (2011 Census) and is among the fastest growing population areas in the U.K. [20], and thus space for living (urban development) is vital.

In our approach, identifying priority areas for ecosystem services, urban development and renewable energy provision together, serves as a tool for optimizing their provision, and for promoting their consideration during the landscape management decision making processes. Finally, in determining priority environmental service areas for Cornwall we can exploit developments in spatial conservation planning that enable the consequences of differentially weighting services to be determined [13], [16]. Because there are no a priori sets of objective rules by which appropriate weightings of different services can be determined, it is important to understand what effect different decisions would have.

Bearing in mind the complex challenges described above that occur while carrying out spatial analyses and prioritisations of environmental services, we address the following fundamental questions: (i) how are the values of key services spatially distributed?; (ii) what are the spatial covariances between services and the consequences for the spatial co-occurrence of services?; and (iii) where are the priority areas (locations where one or multiple service provision is greatest) for environmental service provision? The answers to these questions are crucial to include the ecosystem and environmental service value in landscape management practice.

## Methods

### Data

We built gridded landscape maps of environmental service provision for Cornwall at a resolution of 1×1 km (for a total area of 3,478 km^2^), in each case standardising the variables to a scale of 0 to 100 (see material S1 for detailed processing routines). We considered three distinct cultural ecosystem service values: the tourism value of ecosystems in attracting visitors from distant areas; the recreational value of ecosystems in attracting visitors for leisure; and the aesthetic value of ecosystems to people.

#### Tourism (cultural service)

We determined tourism value using the distance travelled by visitors to natural sites. We used data from the Monitor of Engagement with the Natural Environment survey database [26]. From the original datasets, we selected the 160,376 records for Cornwall during the three time periods available (years of survey 2009, 2011 and 2012). Each record included the geographical coordinates of the place visited and the distance travelled to that particular site. For each grid cell, we accumulated the distance travelled to each site.

#### Recreation (cultural service)

We used the total area of public parks, gardens and golf courses in each grid cell as a measure of its value for recreation. We mapped golf courses based on an initial list of their approximate location [27] and then digitized their extent using aerial images and GIS software (Quantum GIS Bing aerial image plug-in [28]). Likewise, we used data from the National Trust parks and gardens data set [29] and from the English Heritage register of Parks and Gardens [30]. Finally, we merged the three data sources and built a common spatial dataset of publicly accessible parks, gardens and golf course facilities.

#### Aesthetic (cultural service)

Following Casalegno et al. [31], the aesthetic value of each grid cell was measured by counting the number of individual users uploading photographs on the “Panoramio” geo-tagged social media resource (113,686 photographs uploaded by 15,413 users). The methodology proceeds from the premise that images will be captured by greater numbers of people in areas that are more highly valued for their aesthetic attributes; this measure is more appropriate than the number of photographs uploaded, which can reflect the level of activity of individual photographers rather than the overall value placed on a site by visitors.

#### Agricultural value (provisioning service)

Following the basic methodology of Anderson et al. [32] and Eigenbrod et al. [22], as an overall measure of agricultural production we determined the summed gross margins of all major crops and livestock without considering subsidy payments [31]. Agricultural census data at ward level [33], the 100×100 m resolution CORINE land cover map [21], and gross margin estimates from the Farm Management Handbook [34] were used as inputs. Agricultural production was expressed in units of £/ha, processed at 100 m resolution and then resampled at 1×1 km resolution. We improved on the original methodology by computing an averaged agricultural value from 2000 to 2005 (instead of using one year of data); differentiating gross margins according to lowlands, disadvantaged and severely disadvantaged areas [35]; and using more precise input land cover data to achieve a higher resolution.

#### Soil organic carbon (regulating service)

Data on the organic content of topsoil were obtained at a resolution of 1×1 km from the European Commission Joint Research Centre [36]. These data are especially accurate for England, as detailed ground survey verification has been carried out [37].

#### Aboveground carbon (regulating service)

An aboveground carbon map of Cornwall was computed using three different input layers: a 25×25 m resolution tree cover map [38], the CORINE land cover map at 100×100 m resolution [21], and the carbon density conversion tables available from bibliographic references [39]. We extracted all classes including vegetation from the land cover map and merged these data with the tree cover layer at 25×25 m resolution. We then used the conversion table and calculated t/ha for each vegetation class (see material S1 for detailed methodology and conversion values). Finally, we resampled the grid map to a 1×1 km resolution.

#### Flood mitigation (regulating service)

We quantified the flood mitigation capacity of the landscape in terms of its potential downstream drainage and impact on flood risk zones, using an approach similar to that others have proposed [14], [40]. The output flood mitigation capacity is the result of an additive function integrating: a reclassified land cover type (in terms of potential flood mitigation capacity); the water accumulation of each basin cell grid; the slope of the terrain; and the number of buildings affected in flooded risk areas (details in material S1). We used the hydrological modelling algorithm r.watershed [41], [42] available in GRASS software [43] to compute the main input topographic parameters: terrain slope angle, water accumulation (the number of upstream cells from each flood risk cell) and watershed basin distribution extent (for 282 drainage basins). The Advanced Spaceborne Thermal Emission and Reflection Radiometer - Global Digital Elevation Model [44] at ∼23×23 m resolution was used as the input to run the hydrological modelling. The historical flood risk area records in Cornwall were from the Environment Agency (341 warning areas) [45]; and the number of buildings in the flood risk areas was from the vector layer of Edina Digimap [46]. The land cover type was from the CORINE land cover map at 100×100 m resolution [21] supplemented by the forest type map [47] to specify broadleaved, mixed and coniferous forest cover at 25×25 m resolution.

#### Plant production (supporting service)

We used data on the normalized difference vegetation index (NDVI) derived from satellite data as a measure of plant production [48]–[51]. The relationship between plant production and NDVI is well established and documented theoretically and empirically [52], [53]. The sum of positive NDVI values over time is a useful measure for vegetation production [54], [55]. We computed the sum of positive NDVI time series of data from the Moderate-resolution Imaging Spectroradiometer (MODIS) sensors on board NASA's Terra satellite. The spatial resolution of these data is 250×250 m and its temporal resolution is two images per month. We used data from February 2000 to January 2013 [56], merged the tiles per time shoot corresponding to the extent of Cornwall, computed the sum of positive NDVI values, extracted the pertinent study area and rescaled the final map to a 1×1 km resolution grid.

#### Urban development (Living space; other environment service)

Coverage by urbanised areas was determined using the CORINE land cover map [21] at 100×100 m resolution and by merging the land cover classes “continuous” and “discontinuous urban fabric”, “green urban”, “industrial and commercial units”, “port areas” and “airports”.

#### Solar energy production (other environmental service)

We estimated solar energy production in Cornwall as the product in a grid cell of existing solar photovoltaic panel surface area and solar irradiation. To calculate solar panel surface area, we mapped the 18 active solar parks producing photovoltaic energy (installation > = 1 MW and/or > = 1 ha) using the number and approximate location of solar parks from Cornwall Council [57] records and the same method tools as described for recreation. Irradiation data were from the European Commission Joint Research Centre [58] and quantified the yearly average sum of irradiation on an optimally-inclined surface (kWh/m^2^ period 1981–1990) [59].

#### Wind energy production (other environmental service)

Wind energy production was mapped considering all wind turbine sizes from domestic (<18 m height) to small (26–60 m), medium (61–99 m) and large turbines (100–150 m height). We used Cornwall Council records for wind turbine energy production and wind farms [60] and digitized turbine locations and their energy production in MW per turbine. We also added smaller turbines visible on aerial images and not reported in the Council records. For these we took a conservative approach, assuming a minimum production of 0.2 MW per turbine. We identified a total of 251 turbines including both council records and smaller wind turbines detected on aerial images.

### Analysis

The overall patterns of spatial variation within maps were quantified using Moran's I index [61]. Moran's I index approaches a value of 1 when there is a high degree of clustering, whereas values approach zero for disperse and random distribution patterns. We determined the spatial covariance between each of the environmental service layers in Cornwall using the Clifford Richardson Hemon correlation method (CRH) [62], [63] on rank transformed inputs to correct statistical significance for spatial autocorrelation. We tested the stationarity of spatial covariance patterns (i.e., changes of correlation amplitude and direction according to space) by dividing Cornwall into four geographical zones (coastal, west, central and east Cornwall) and compared the spatial correlation within zones. In addition, results were corrected for multiple test significance using Benjamin-Hochberg corrections [64].

Co-occurrences between service layers were quantified by iteratively selecting from the input layers sample areas that contain a given percentile of the value of each layer, from the minimum to maximum valued grid cells. In other words, we recoded the service layers originally standardized in continuous values from 0 to 100 into 100 binary maps, each one having a threshold corresponding to percentiles running from 1 to one hundred. We then quantified the percentage value of the other co-occurring services within the one hundred recoded binary maps valued 1.

We used the software package R [65] for statistical analyses (see material S1 for detailed processing routines), and Zonation [66], [67] to carry out the prioritisation analysis. Zonation is a spatial conservation planning tool that produces priority ranking maps. It was originally created for biodiversity conservation but it has also been used for environmental service prioritisation purposes [16], [68]. The Zonation algorithm addresses the “maximum utility problem”, that is, maximising the retention of valuable areas in terms of biodiversity (or ecosystem services in our perspective) [4]. Zonation works on raster grid input data (species distribution maps and/or environmental service maps). The algorithm first sums the value of each environmental service layer to be prioritised and recursively discards the least valuable cells and recomputes the sum until all cells are removed. The last cells to be removed have the highest ranking while the first removed have the lowest ranking, allowing the production of a continuous value ranking map. Several removal rules are available; all are based on the minimization of marginal loss (i.e. relative contribution of each cell to total conservation value) of biodiversity. In our analyses, we do not focus on biodiversity conservation but on the value of the landscape for services provision and we aim to prioritise the landscape accordingly. Several criterion for calculating the marginal loss of biodiversity/environmental services are available. We used the so called basic core area zonation [66], [67], which allocates high values to areas where one or multiple layers have an important occurrence. This specific removal rule produces a high rank in areas where high values occur within a single input layer. The basic core area rule was preferred to the alternative benefit function rule, which produces high values where input layers occur simultaneously at potentially low occurrence levels [67]. The benefit function rule is more appropriate for promoting the conservation of all species in conservation prioritisation of biodiversity [69]. We also parameterised the algorithm with a “no edge removal criterion”. This criterion allows, at each subsequent iteration, the remove of cells from anywhere in the available landscape. This technique is different to the “edge removal criterion”, which forces the algorithm to remove cells from the edges of the remaining landscape at each iteration and produces connected priority areas. Edge removal criterion are more suitable for biodiversity conservation proposes where clustered areas are valued. For environmental services provision prioritisation however, there is no *a priori* reason to connect clusters of highly ranked landscapes.

Zonation can be parameterised with positive or negative weights for each layer to increase or decrease the relative value of a service. This allows the prioritisation to be skewed toward a particular service of interest. Since the weight allocated to inputs determines the overall output prioritisation map, we carried out analyses using unweighted and weighted input layers. The weighted prioritisation analysis was carried out on groups of equally weighted services. The groups were: (i) cultural services: recreation, aesthetic and tourism; (ii) energy-related services: solar and wind; (iii) carbon stock related services: above and below ground carbon stocks; and single services weighted independently: (iv) flood mitigation, (v) agricultural production, (vi) plant production, and (vii) urban development. Since the weighting factor is a major determinant of the output prioritisation results, we carried out a sensitivity analysis of 24 weighting thresholds running from −100 to +100 (±2; 5; and 10 to 100 by step 10). The selection of the appropriate weight is carried out by (i) plotting weight thresholds versus the value of each service and the cumulative value of the co-occurring services; and (ii) selecting an appropriate weight threshold to increase the provision of a specific service without compromising the overall provision of the other complementary services. As a result of the sensitivity analysis, we selected 14 thresholds positively and negatively to weight the seven groups of services.

## Results

### Spatial variation

**Figure 1:**
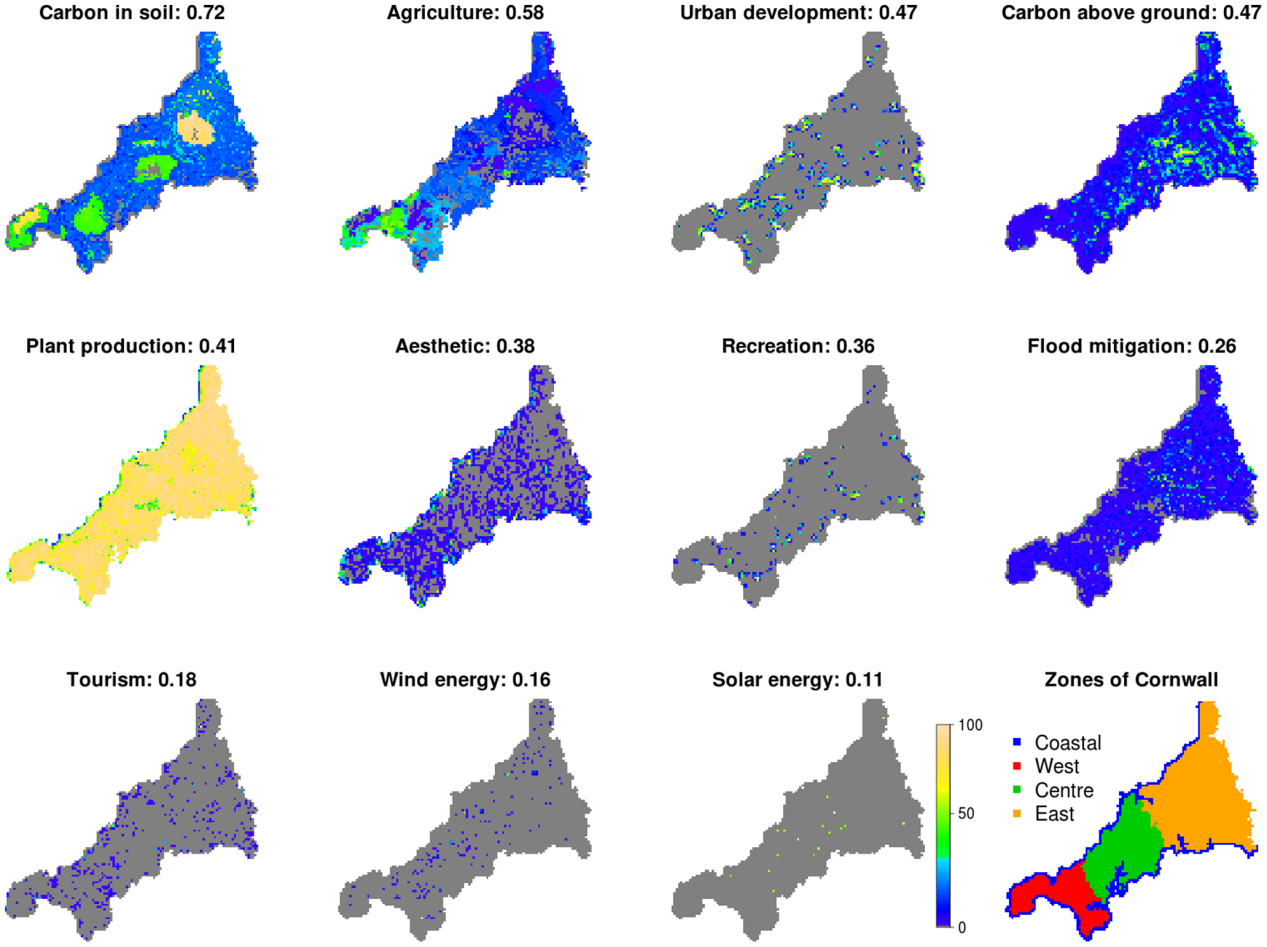
Spatial distribution of environmental services in Cornwall and the different zones of the region. Grey areas correspond to a minimum service value equal to zero.

**Figure 2:**
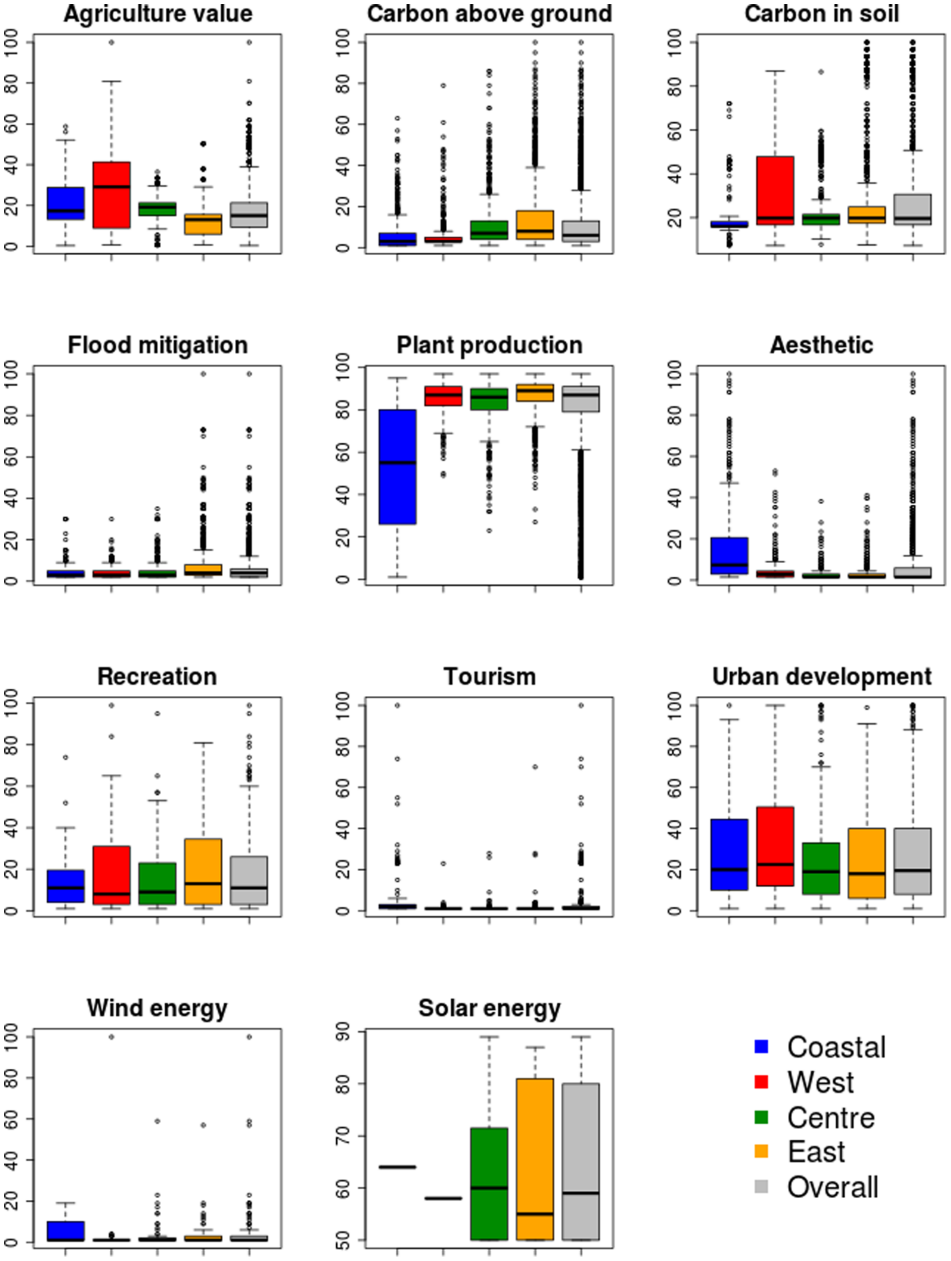
Zonal statistics of environmental services in Cornwall and within zones thereof. Shown are the median value (central line), upper quartile (edges of boxes), maximum and minimum values excluding outliers (whiskers) and outliers (dots).

Virtually all of the environmental services studied exhibit marked spatial variation across the region (Figure 1). Some show dispersed patterns producing low Moran's I indexes (e.g. energy related services, cultural services, and flood mitigation in Figure 1). Others have Higher Moran's I indexes as a result of clustered patterns (e.g. agriculture, below and above ground carbon stocks, urban development and plant production in Figure 1). The extremes of such patterns are solar and wind energy layers on the one hand (dispersed) and soil carbon and agriculture on the other hand (clustered), while the remaining layers form a continuum between the extremes. The most valuable areas within Cornwall (Figure 2) are found in the west zone for agriculture, in the wooded areas of the central and east zones for above ground biomass, and in acid peat soils in the upland areas in the west and east zone for soil carbon. Plant production is more evenly distributed with the exception of low values in northern coastal zones. Tourism and aesthetic values have peaks in coastal zones, while recreation and urban development are more homogeneously distributed. Renewable energy layers have the lowest coverage and limited distributions; excluding outliers, the highest values are found in coastal areas (wind energy) and in east Cornwall (solar energy).

### Spatial covariance

We tested 55 correlations between services (eleven service interactions) in each of five different geographical extents (overall study area and within the four zones of Cornwall: Figure 1): 32 of the 55 spatial interactions between services were significantly correlated across the overall study area and 38 were significantly correlated in at least one geographic zone (Table 2). Correlations were not stationary and statistical significance varied depending on the zone (see standard deviations in Table 2). Within the four zones of Cornwall, of the 220 correlations tested (55 service interactions ×4 zones), 83 were significant (24 in coastal zone, 19 each in west and central Cornwall, and 21 in east Cornwall). Energy production was found either not to be significantly correlated with other services (solar) or to have low correlation coefficients (wind). The strongest negative correlations were found between soil carbon and agriculture or urban development; flood mitigation and urban development; and plant production and urban development or aesthetic values. The strongest positive correlations were between agriculture and plant production; carbon stocks, plant production and flood mitigation; cultural services; and cultural services and urban development.

**Table 2:** Correlations between spatial variation in environmental services in Cornwall and within zones thereof.

	Agri	Car A	Car S	Miti	Prod	Sol	Aest	Recr	Tour	Urban	Wind
Agri		ns	E*** (3)	C*** (1)	C*** (2)	ns	**W** * **(2)**	**C** * **(1)**	C*** (3)	Ce*** (4)	ns
Car A	ns		C*** (3)	C*** (5)	C*** (4)	ns	O* (2)	C*** (5)	**O** * **(1)**	Ce*** (3)	ns
Car S	−0.30±0.21	0.13 ±0.16		O*** (5)	C*** (4)	ns	O*** (1)	W* (4)	W*** (3)	W** (3)	O*** (1)
Miti	0.03±0.12	0.30±0.13	0.20±0.10		C*** (3)	ns	O*** (1)	**C** * **(1)**	O*** (3)	Ce*** (5)	ns
Prod	0.10±0.15	0.30±0.23	0.16±0.27	0.25±0.22		ns	O*** (2)	C** (1)	O*** (4)	W*** (4)	O*** (1)
Sol	ns	ns	ns	ns	ns		ns	ns	ns	ns	ns
Aest	**0.10±0.03**	0.06±0.01	−0.02±0.12	−0.06±0.09	−0.13±0.11	ns		O*** (5)	C*** (5)	C*** (5)	ns
Recr	**0.02±0.09**	0.15±0.02	−0.06±0.10	**0.02±0.05**	0.00±0.09	ns	0.13±0.01		C*** (4)	C*** (5)	**O** * **(1)**
Tour	0.09±0.05	**0.03±0.02**	−0.08±0.09	−0.07±0.04	−0.10±0.08	ns	0.27±0.11	0.14±0.05		C*** (5)	ns
Urban	0.17±0.06	0.09±0.08	−0.12±0.13	−0.16±0.04	−0.16±0.17	ns	0.24±0.06	0.25±0.10	0.34±0.07		ns
Wind	ns	ns	0.05±0.02	ns	0.05±0.13	ns	ns	**−0.03±0.01**	ns	ns	

Lower left matrix: mean value and standard deviation of correlation amongst the zones. Bold values highlight those correlations that are non-significant when p-value is corrected for multiple tests and spatial autocorrelation. Upper right matrix: zone where the correlation was maximum (O: Overall Cornwall; E: East; W: West; C: Central; ns: not significant);

*p<0.05;

**p<0.01;

***p<0.001.

P-values are corrected for spatial autocorrelation (CRH method). In parentheses the number of zones in which the correlation was significant. Agri: agriculture; Car A: carbon above ground; Car S: carbon in soil; Miti: flood mitigation; Prod: plant production; Sol: solar energy; Aest: aesthetic value; Recr: recreational value; Tour: tourist value; Urban: urban development; Wind: wind energy.

Correcting correlations for both spatial autocorrelation and multiple tests increased the p-value of six correlations to a non-significant level (p>0.05). From the 55 spatial interaction between services, 33 where significantly correlated at least in one geographic zone. From the overall 220 correlations tested within the different zones, the number of significant correlations dropped from 83 to 77 (see bold values in Table 2). The six non-significant correlations were: aesthetic and agriculture (in two zones); aesthetic and recreation; carbon above ground and tourism; flood mitigation and recreation; wind and recreation. Between the 33 significant correlations corrected for multiple tests and spatial autocorrelations, 17 of them have strong spatial non-stationarity (standard deviation is higher than the mean value of correlation coefficient between zones in Table 2).

### Co-occurrence between distribution patterns

**Figure 3:**
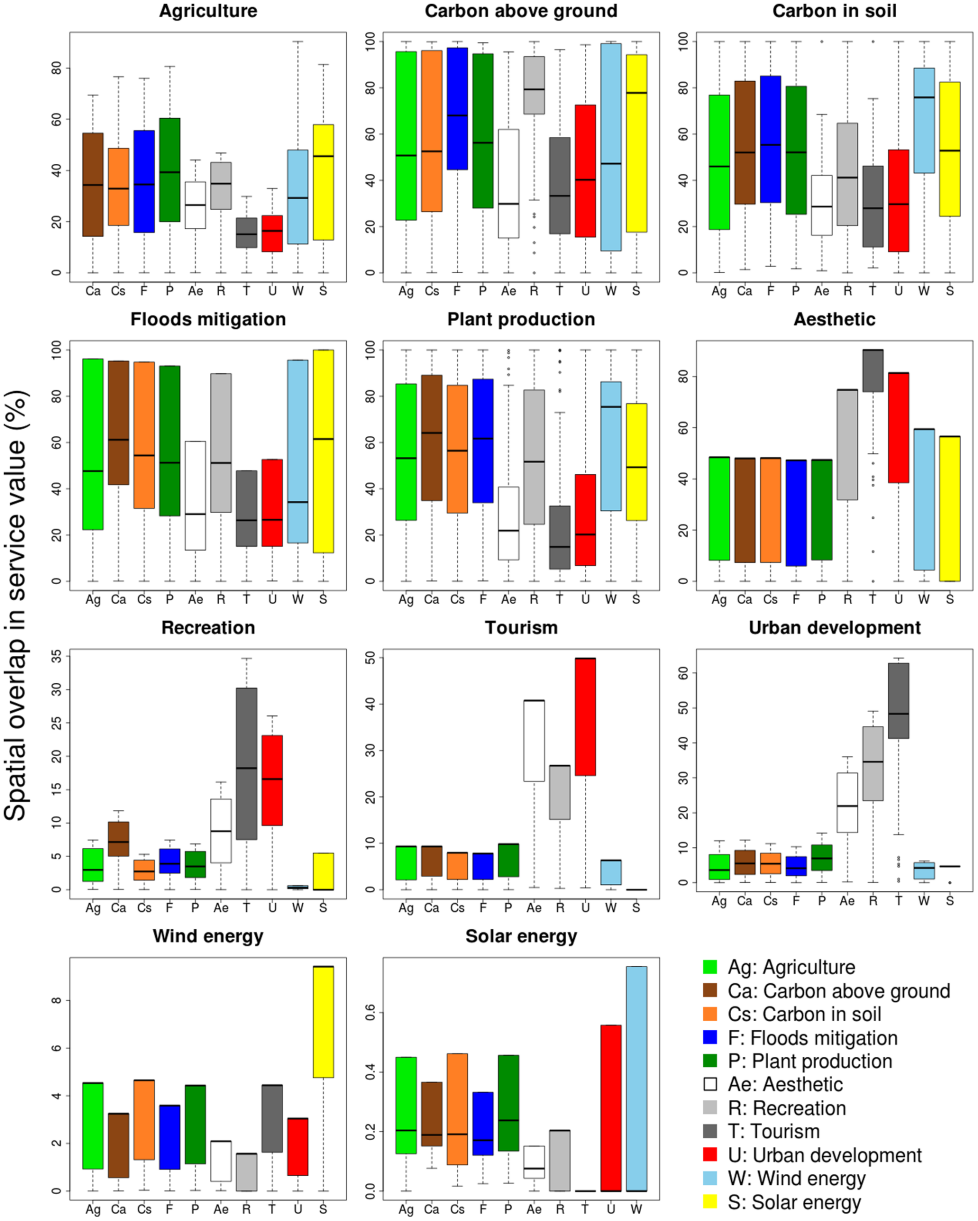
Co-occurrence of environmental services. In each panel is quantified the spatial overlap between a main service and its co-occurring services. The y-axis represents the percentage spatial overlap of the overall co-occurring service value available within the main service: maximum distribution area (top segments or bullet outliers of whisker histograms); the area excluding the lower quartile valued cells (top edge of coloured histogram), the area from the median to the top valued cells (black horizontal lines); the higher quartile cells valued areas (lower edge of coloured histograms), and the area available within the maximum value grid cells (lowest segments or bottom outliers of histograms). Outliers and maximum values are not visible when overlapping with quartile upper boundary.

The complexity of interactions between environmental service distributions generates multiple different co-occurrence patterns. Environmental services co-occured with ecosystem services. In Figure 3, the top segments or bullet outliers of whisker histograms show the current overlap between environmental services (full results in Figure 1 of material S1). Due to their limited distributional extent, renewable energy layers overlapped with a negligible proportion of other services (<1% for solar energy and <5% for wind energy). Despite the societal conflicts concerning the development of wind farms and their impact on landscape aesthetics, we found less than 2% of the overall aesthetically valuable areas co-occurring with wind energy production sites (less than 2% for recreation and less than 4% for tourism). The occurrence of solar energy production also overlaps with less than 0.2% of the overall value of each cultural service.

Cultural services had maximum co-occurrence with each other and with urban development: recreation overlapped with 35% of the overall value of tourism, tourism overlapped with 50% of the overall value for urban development, and aesthetic value overlapped with 90% of the overall tourism value. Urban development overlapped with, respectively, 65%, 49% and 35% of tourism, recreation and aesthetic services. Overlaps with all other services were less than 15%. Land with flood mitigation value had a lower overlap with aesthetic (60%), tourism and urban development (∼50%) than for other services (>90%).

Considering the most valuable areas per service (top quartile in Figure 3) and their corresponding dominant overlapping layers, grid cells with flood mitigation values of 75 or more co-occurred with aboveground carbon (followed by carbon in soil, recreation and plant production); agriculture and aboveground carbon co-occured with recreation (∼25% and ∼70% of the overall recreation value respectively); and soil carbon overlapped with ∼45% of the wind energy service. Average to top plant production landscapes (Figure 3 black horizontal lines of whiskers histograms) had lower overlap with aesthetic, tourism and urban services (<25%) as compared to other services (∼50 to ∼75%). In highly valued agricultural areas (bottom edge of whiskers in Figure 3), we found low co-occurrence with solar energy (10%); when also considering low value agricultural landscapes (cell grid value >0) the overlap increased to 80%.

### Priority areas

**Figure 4:**
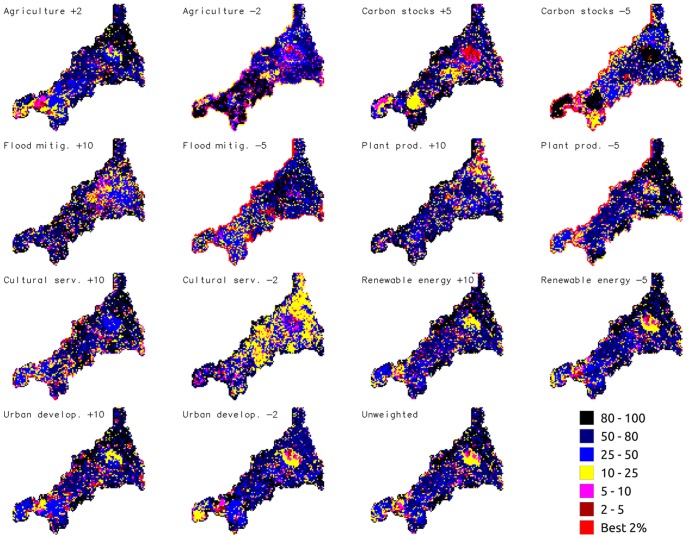
Priority maps for environmental services in Cornwall using different weighting strategies. Blue to black (minimum value) are the least valuable areas and yellow to red the most valuable. Weight levels stated in maps titles.

Using the Zonation approach, we first constructed priority maps for unweighted environmental services. The resulting map (Figure 4 - panel unweighted) highlights sparse and clustered high value zones throughout Cornwall. The largest extent of high priority landscape was found in west, inland-east and coastal Cornwall, while large extents of low priority landscape were found mainly in north-east and central Cornwall.

**Figure 5:**
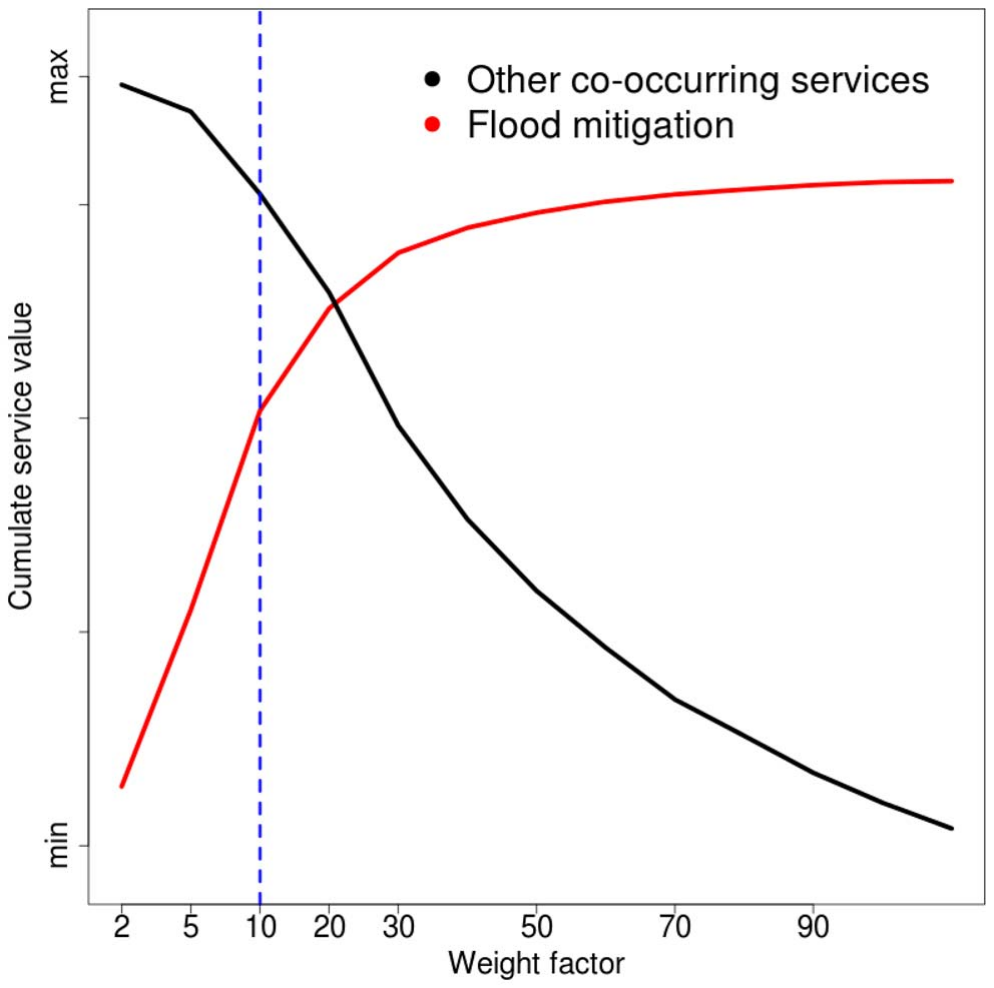
Sensitivity analysis for weight selection for flood mitigation. For each weight the cumulative value of flood mitigation prioritisation performance curves (red line) and the cumulative value of all the other service performance curves (black line) are shown. A weight equal to 10 (dotted line) defines a prioritisation map appropriate to maximise the conservation of areas for flood mitigation purposes without compromising the overall value of the other services. Lower weights do not force the zonation algorithm to preserve areas with strong flood mitigation values while higher weights markedly decrease the overall cumulative value of other services.

To determine the sensitivity to weighting environmental services, overall we computed 168 (24 weights ×7 groups) separate prioritisation scenarios. A complete list of sensitivity curves for weight selection is provided as (Figure 2 in material S1). Weights of between +10 and −5 prioritise target services and avoid excessive loss of co-occurring services. As an example, a weight of 10 is appropriate for positively prioritising flood mitigation without compromising the overall cumulative value of other services (Figure 5). Weights higher than +10 would have resulted in a priority ranking map closely related to the input flood mitigation map. In this case, locations where the other services provision value was high would be excluded from the priority areas. In Figure 5, a lower weight (e.g. 2 or 5) did not significantly increase the overall value of flood mitigation, and higher values (20 or higher) severely impacted the overall value of the co-occurring services.

**Figure 6:**
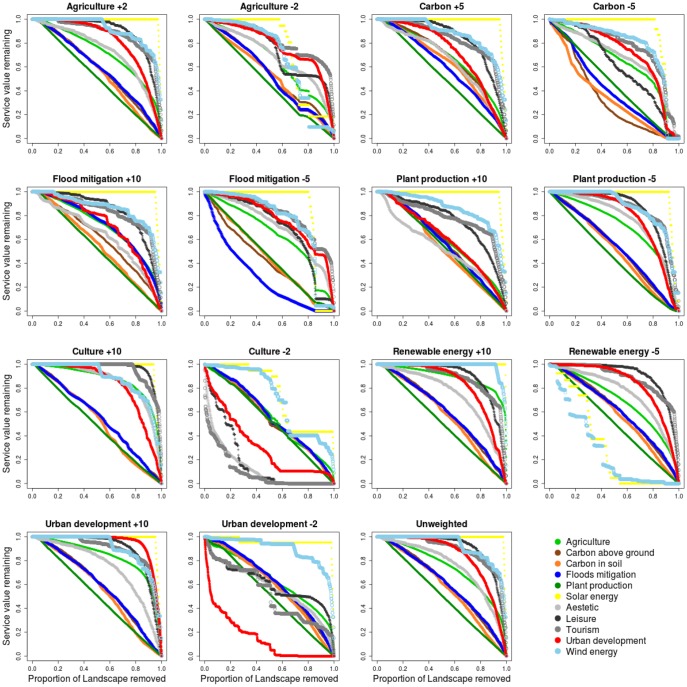
Performance curves corresponding to prioritisation simulations with different weighting schemes. In these figures the x-axis represents the proportion of the total land area removed, starting with the areas with the lowest value for environmental services. The lines show the proportion of the total service value for each category remaining. Symbols below the 1∶1 line show that the service is associated with regions with low prioritisation under this weighting scheme, and lines above the 1∶1 line show that it is associated with regions with a high prioritisation.

Figure 4 shows maps representing different weighting scenarios, using both positive and negative weights. The same weighting scenarios are represented in the relative performance curves (Figure 6). Energy related services were included within priority areas in most of the scenarios, even when a high proportion of lower value landscape was removed from prioritisation. Plant production, carbon stocks and flood mitigation were lost from priority areas more consistently, as prioritisation focuses on the highest value areas. A positive weighting for energy related layers or urban development favoured the provision of such layers without compromising the co-occurring layers as compared to the unweighted analysis (i.e. the positively weighted energy prioritisation map is similar to the unweighted map). In contrast, increasing the weight of flood mitigation had a much stronger impact on the other services (e.g. urban development and cultural service performance curves) as compared to an unweighted analysis. Negative weights provoked the most abrupt changes as compared to positive or unweighted analysis, as is evident both from the output maps and in the performance curves (i.e. culture, agriculture, carbon stocks and urban development in Figure 6). For example, a prioritisation strategy that excludes urban areas will involve losing areas of leisure and tourism value from the priority areas, and one that excludes high value agricultural areas will lead to a loss of most other services compared to an unweighted scheme.

## Discussion

Studies that document the fundamentals of how environmental services are spatially distributed, and how they covary and coexist are scarce, particularly when considering regional scales. Here we have highlighted the patterns of covariance and co-occurrence in those distributions, and the distribution of priority areas for environmental service provision, whilst also addressing the key challenges for regional relevance. We have underlined the need of regional analysis to address the issues of using appropriate spatial extents and resolutions, avoidance of heavy reliance on benefits transfer, focus on significant services, application of appropriate prioritisation tools, and inclusion of both ecosystem services and other environmental services.

The key findings shown in this analysis are as follows: (i) the provision of environmental services (renewable energy and urban development) spatially overlap with the provision of ecosystem services (Figure 3); (ii) there are two main patterns of service distribution (Figure 1): dispersed (agriculture, carbon stocks related, flood mitigation and plant production) and aggregated services (cultural, energy related and urban development); and (iii) more than half of the services are spatially correlated and there is high non-stationarity in the spatial correlation between services (Table 2). Correlations were negative between soil carbon, agriculture and urban development, and positive between agriculture and plant production, between carbon stocks, plant production and flood mitigation, within cultural services, and between cultural services and urban development. The highest correlations were found between urban development and cultural services, and to a lesser degree between ecosystem services. Finally (iv) priority areas for the provision of overall service value were located in coastal zones, and in west and east-inland Cornwall (Figure 4).

From a methodological point of view, our analyses suggest that using analytical tools, such as the Zonation prioritisation algorithm, weighting factors between services need to be carefully tested and selected. For instance a weight factor of 2 or 5 for floods mitigation would not provoke the expected increase in its prioritisation scores (Figure 5). Processing a zonation ranking map with an overestimated weighting produces an unbalanced prioritisation map where only the weighted input layer is considered as priority and the other layers importance are neglected. This is a fundamental issue for scientific knowledge transfer to landscape management. Moreover, our results suggest the importance of including other environmental services in ecosystem service studies since these sets of services often overlap and must be managed in conjunction with ecosystem services (e.g. cultural services with urban development, renewable energy with agricultural production, plant biomass and soil carbon stock in Figure 3).

### Consequences for the distribution and spatial co-occurrence of services

Our analyses are useful for understanding which co-occurrences between the distributions of services are potentially useful for developing higher value landscapes. Negative correlations imply that different services are spatially segregated, and hence different areas supply different services. This may be because some areas are inherently suited to supplying one service. For example, in Cornwall upland soils are more suited to carbon storage and less agriculturally productive (in Figure 1: high values of carbon, visible as clear brown areas in east Cornwall in the soil carbon map, coincide with low value agriculture landscapes visible as grey and blue zones on the agriculture map). Alternatively, different areas may supply different services, because exploitation of one service is occurring at the expense of others (for example urban development and agriculture in Figure 3). Positive spatial correlations between ecosystem and environmental services highlight ecosystems that provide multiple services. Areas supporting multiple services may be the source of win-win situations, where ensemble management or conservation of an ecosystem provides multiple benefits (for example woodland may provide both flood mitigation and carbon storage), or alternatively may highlight areas of conflict or trade-offs where maximising provision of one service may occur at the expense of others (urban development contrasting with agriculture or flood mitigation). This last pattern is visible in Figure 1 and 2, where both agriculture and urban development have high values in west Cornwall. This is also spatially shown in Figure 4. While looking at positive weights for urban development in west Cornwall (Figure 4: urban development +10 panel), the yellow zone and adjacent red area indicate highly valuable landscape for urban development, which is contrasting with the high agricultural productivity scores (see the same area for the positively weighted map of Agriculture +2 panel in Figure 4).

Within areas supporting multiple services, we found the co-occurrence of renewable energy layers (Figure 3 - higher values of Solar energy in the Wind energy panel and high values of Wind energy in the Solar energy panel); and the co-occurrence between energy layers with soil carbon stocks and appropriate flood mitigation managed landscapes (Figure 3 - energy layers in Carbon in soil panel and solar energy in Floods mitigation panel). We also found co-occurrence between high value areas for carbon stocks in vegetation, recreation and flood mitigation (Table 2; Figure 3: Carbon in soil panel). These last sets of services are well suited for ensemble management.

Our results show (Figure 3) that in Cornwall the current distribution of services such as aesthetic-cultural services vs wind farms or solar parks have been carried out without critical overlap between landscape development opportunities from an environmental perspective. Currently, for example, solar parks are located in low value agricultural areas and we found clustered overlapping areas of development for renewable energy and carbon stocks on one side and agriculture and recreation or floods mitigation and carbon storage on the other side. Such co-occurrences are win-win situations and do not require trade off choices in a future perspective of sustainable development. Considering renewable energy, the management of the overlap between solar parks and agriculture is complex, while wind turbines currently co-exist with pasture lands. Trade-off between agricultural production and solar parks may be overcome in the future by using suspended photovoltaic panels fully integrated in agriculture [70].

The aesthetic value of landscapes, and how they are impacted by the presence wind turbines and solar parks represents an on-going and dynamic debate, which, given the increasing demand for renewable energy is unlikely to abate. The analytical approach we developed is well suited for the analysis of such constraints and the selection of appropriate land management solutions. Different weighting schemes may be used to explore the dynamic nature of attitudes towards the aesthetic value of wind turbines and solar parks. Future development of this approach may incorporate the potential for such land uses to impact the aesthetic value of landscapes on a scale beyond the installations themselves.

### Prioritizing environmental services

The complexity and diversity of challenges involved in integrating environmental services into land use planning and decision-making suggest the need to develop a set of prioritisation scenarios that are capable of generating more than a single optimal solution. Prioritization strategies will depend on the political, technological and social context, as well as the relative values allocated to different services. For this reason, we first created a map of unweighted environmental and ecosystem services priority for maximising the provision of the ensemble of layers (Figure 4: Unweighted panel); and secondly processed a set of modelling outputs by adjusting these service weights (Figure 4). Our aim was better to understand the implications of prioritising one service and its impacts on the others and provide decision makers and stakeholders with a tool for discussing solutions. Output prioritisation maps can inform decision making at scales of 1 km or aggregations thereof (watershed basin or administrative unit). In particular, our analyses can identify the potential for balancing two different management approaches (Figure 6): “spatial segregation of services”, where the management of landscapes areas is prioritised to maximise the value of one or more services at the expense of others, or “ensemble management”, where management for many services is balanced to gain the maximum total value.

In Figure 6, virtually all points lie above the 1∶1 line, implying that there is limited scope within the region for spatial segregation of environmental services at the spatial scale used in this study. Spatial segregation is found in: (i) Urban development: in the weighting scenario where it has a negative weighting (Figure 6: Urban development −2 panel) it appears that ensemble management of other services could occur in priority areas excluding much of the urban area of the region, without a high loss of other service value. However, if urban areas are excluded, some areas of aesthetic, leisure and tourism services will also lie outside the priority areas. (ii) Renewable energy: negative weighting of renewable energy (Figure 6: Renewable energy −5 panel) will not impact the complementary services (curves similar to the unweighted scheme) because of the limited spatial extension of renewable energy production. (iii) Flood mitigation: negative weights introduced for floods mitigation affect above ground carbon (Figure 6: Flood mitigation −5 panel). (iv) Plant production: positively weighting plant production (Figure 6: Plant production +10 panel) decreases the overall value of aesthetic value: we found negative correlation between those two services (Table 2). (v) Carbon: (Figure 6: carbon −5 panel) in the case where it gets a negative weighting, land with a high value for carbon storage can be excluded to some extent for management for other services. In contrast, even when agriculture is given a negative weighting, high value agricultural areas are still included in prioritisation regions, suggesting that ensemble management of environmental services within the region must include provision for agricultural services.

Once determined priority areas, decision makers could proactively manage landscape for promoting their maintenance. Wildlife conservation has mainly focused on the implementation of protected areas for biodiversity conservation purposes. Recent studies have shown that protected areas might not contain and thus effectively sustain ecosystem services [71].

## Conclusions

Our analyses identify priority areas for maximising the overall value of key services provided within a given region. By varying the weights allocated to different services, priority areas can be identified that correspond to different sets of values ascribed to services. Such priority areas could be the focus for management and policy at the regional level, ensuring that planning decisions balance the requirements for ecosystem and environmental services with purely economic concerns. At the level of national to international (European) policy, strategies for sustainable management of landscapes have been discussed. Those include, as an example, payments for carbon sequestration to alleviate trade-off between services [13], and reform of subsidies to the agricultural sector [9], [72]. The choice of services to include within such an analysis, the identification of appropriate weights for different services, and the development of suitable policy responses is a subjective process, and should be driven by the requirements of communities [2], [73] rather than the availability of data. Our findings stress the importance of including both strict ecosystem services, and environmental services that may be independent of the functioning of ecosystems (such as renewable energy production and space for residential and industrial development) within the same decision making framework. We conclude the importance of including a wide range of services linked to regional sustainable development in the landscape management process, not restricted to ecosystem services.

## Supporting Information

Material S1 **Supporting figures.**
(PDF)
