# Supplementary material for: Regional Scale Prioritisation for Key Ecosystem Services, Renewable Energy Production and Urban Development
Source: PLoS One. 2014 Sep 24;9(9):e107822. doi: 10.1371/journal.pone.0107822 (PMC4175084; doi:10.1371/journal.pone.0107822)
Supplement: Material S1 — Supporting figures. (PDF) [file pone.0107822.s001.pdf]

S1 - Supporting information

Figure 1.Co-occurrence between distribution patterns

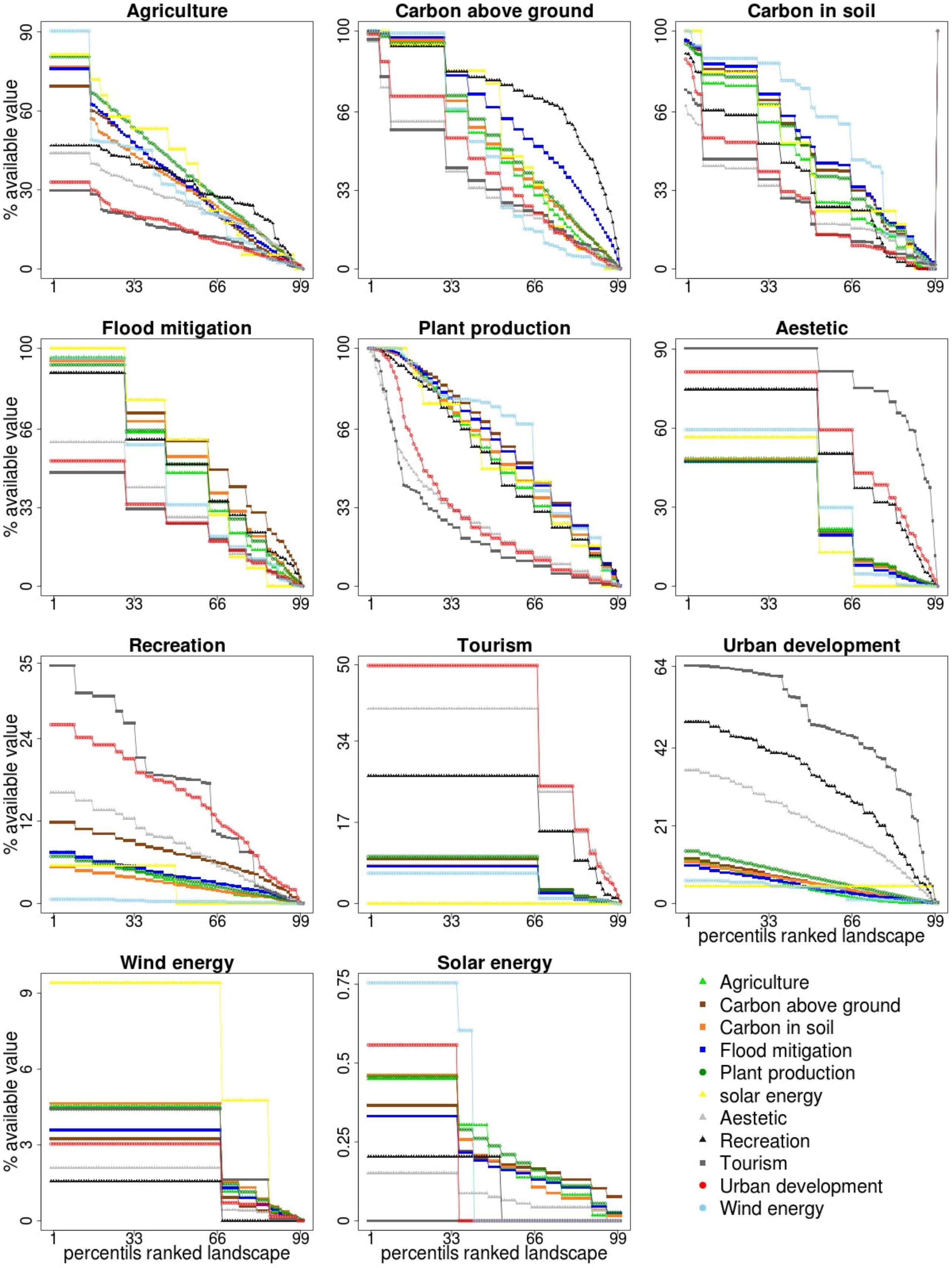

Figure 2: Sensitivity curves for weight selection. In blue thresholds used in the Zonation analyses.

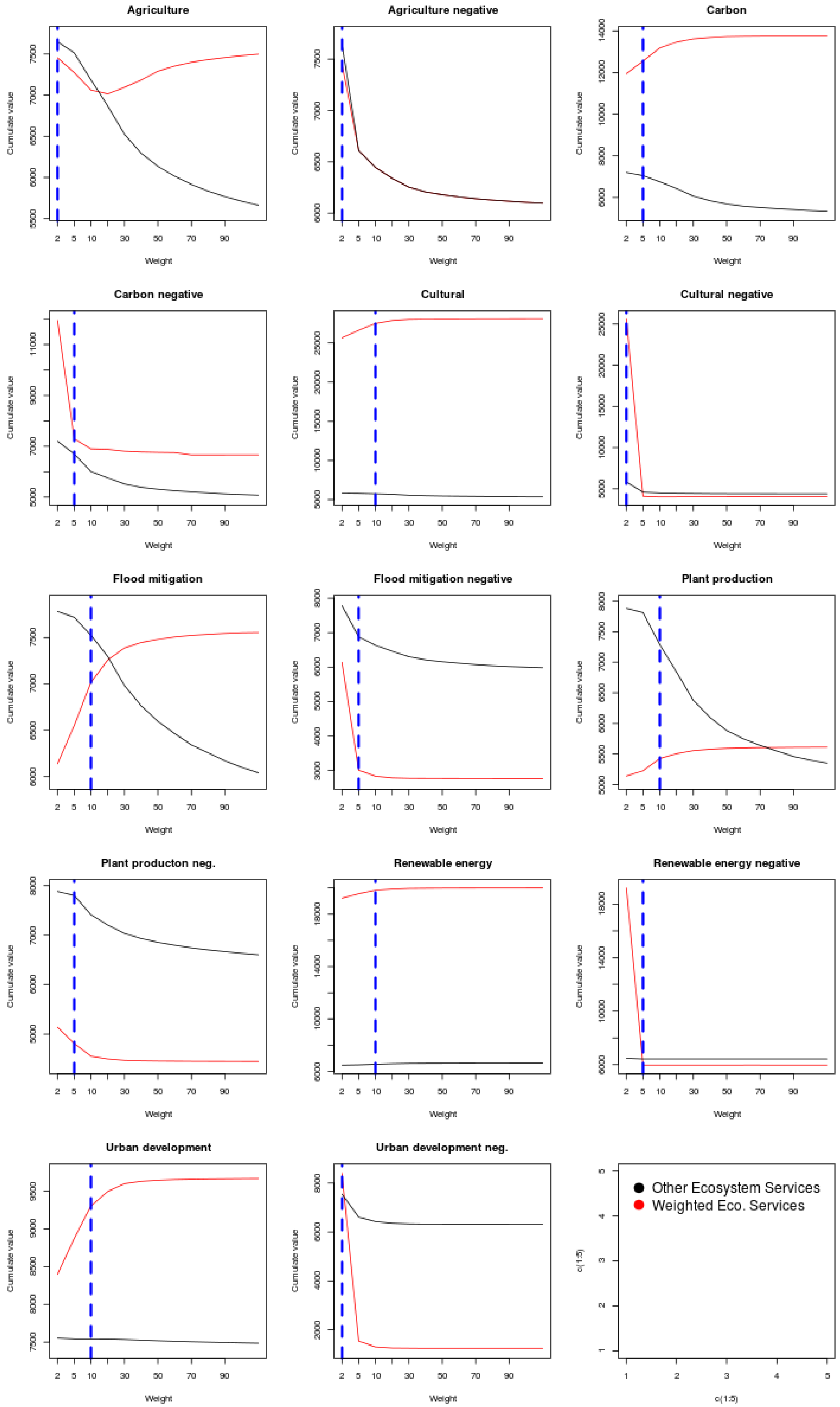

Agriculture
Agriculture negative
Carbon
Carbon negative
Cultural
Cultural negative
Flood mitigation
Flood mitigation negative
Plant production
Plant producton neg.
Renewable energy
Renewable energy negative
Urban development
Urban development neg.
Cumulate value
Weight
Other Ecosystem Services
Weighted Eco. Services
c(1:5)

Mapping ecosystem services and environmental services.
Bash Scripting routine using gdal/ogr libraries, R and Grass.
http://grass.osgeo.org/
http://www.gdal.org/
http://www.r-project.org/

###
ALL OUTPUT data have a resolution of 1000m, geographic progection EPSG:22770 and extent : 133504.5368056825 5614.2066254507517442 246504.5368056825 118614.206625450751744
pixels values run from 0 to 100
All data used in this procedure are open access and dowloadable from the web sites specified below. If web links expired, request data via email
Once processed maps, run the
######### ENGLAND REGIONS administrative borders
storage : ~/downloads/england_nuts2_2001.shp
source : EUROSTAT http://www.ons.gov.uk/ons/guide-method/geography/beginner-s-guide/eurostat/index.html
proj details: EPSG:27700

######### ENGLAND WARD administrative borders
filename: ~downloads/ward98.shp
source : DEFRA https://geoportal.statistics.gov.uk/geoportal/catalog/main/home.page , if web link expired, data can be requested by email
ESRI shape file 8502 fetures
xMin,yMin 131950.41,11073.5 : xMax,yMax 655989.00,675000.00
EPSG:27700 or +proj=tmerc +lat_0=49 +lon_0=-2 +k=0.9996012717 +x_0=400000 +y_0=-100000 +ellps=airy +datum=OSGB36 +units=m +no_defs

######## DEFRA ward level June Agricultural survey for England
storage : ~/EcoServ/agriculture/INPUT/AJS_ward"year"/"year"nuts_all_levels.zip
source : http://www.defra.gov.uk/ , if web link expired, data can be requested by email
1990nuts_all_levels.zip
1995nuts_all_levels.zip
2000nuts_all_levels.zip
2002nuts_all_levels.zip
2005nuts_all_levels.zip
Eng_2010_JC_Data_by_20km_Grid_Square_for_Public_Use.zip
Original file format .xls compressed as .zip ;
xls where coverted to .csv and stored at: ~/agriculture/INPUT/AJS_ward"YEAR"/*.csv where YEAR = 1990 to 2005

######### EUROPEAN LAND COVER
Corine Land Cover 2000
filename: g100_00.zip
source : http://www.eea.europa.eu/data-and-maps/data/corine-land-cover-2000-raster-1/
proj details: EPSG:3035
geoTIFF file at 100m spatial resolution
storage : ~~/downloads/g100_00.tif

Corine Land Cover 2006
filename: g100_06.zip

source : http://www.eea.europa.eu/data-and-maps/data/corine-land-cover-2006-raster-1/
proj details: EPSG:3035
geoTIFF file at 100m spatial resolution
storage : ~/EcoServ/agriculture/INPUT/CLC/CLC06/g100_06.tif

######### LESS FAVOURED AREAS
filename: 772010_maglfa_shp.zip ; contains maglfa.shp
source : http://magic.defra.gov.uk/datadoc/metadata.asp?dataset=32
proj details: EPSG:27700 or   +proj=tmerc +lat_0=49 +lon_0=-2 +k=0.9996012717
+x_0=400000 +y_0=-100000 +ellps=airy +datum=OSGB36 +units=m +no_defs
ESRI shape file 2609 fetures
In layer spatial reference system units : xMin,yMin 83107.2,5336.42 : xMax,yMax
500843.11,641671.19
storage : ~/EcoServ/agriculture/INPUT/LEAST_FAV/maglfa.shp

ALL OUTPUT data have a resolution of 1000m, geographic progection EPSG:22770 and extent :
133504.5368056825 5614.2066254507517442 246504.5368056825 118614.206625450751744
pixels values run from 0 to 100

##
###############
##
###############
# Prepare working environment:
GRASSDB=~/grassdb_AGR
LOCATION=LAEA_AGRI
MAPSETS=GB
OUTPUT=~/output_maps
INPUT=~/agriculture/INPUT

mkdir -p $GRASSDB/$LOCATION
mkdir ~/downloads
mkdir -p $OUTPUT
mkdir -p $INPUT

##
########
#SETTING A GRASS WORKING ENVIRONMENT
N.B. be sure you have the following files in the grassdb folder:
# - create_location.sh download at http://spatial-ecology.net/dokuwiki/lib/exe/fetch.php?
media=wiki:create_location.sh.zip and store here ~/script/.
# - g100_00.tif
~/grassdb_AGR/g100_00.tif
cp ~/downloads/g100_00.tif $GRASSDB/.
cd $GRASSDB
bash ~/script/create_location.sh   g100_00.tif $LOCATION $GRASSDB

Setting GRASS variables for GRASS bash job
echo "LOCATION_NAME: $LOCATION"   > $HOME/.grassrc6
echo "MAPSET: PERMANENT"   >> $HOME/.grassrc6
echo "DIGITIZER: none"   >> $HOME/.grassrc6

```
echo "GRASS_GUI: text"                    >> $HOME/.grassrc6
echo "GISDBASE: $GRASSDB"      >> $HOME/.grassrc6

# path to GRASS binaries and libraries:
export GISBASE=/usr/lib/grass64
export PATH=$PATH:$GISBASE/bin:$GISBASE/scripts
export LD_LIBRARY_PATH="$GISBASE/lib"
export GISRC=~/.grassrc6
export GIS_LOCK=$$ # use process ID (PID) as lock file number:
export GRASS_PNGFILE=/tmp/grass6output.png # settings for graphical output to PNG file
(optional)
export GRASS_TRUECOLOR=TRUE
export GRASS_WIDTH=900
export GRASS_HEIGHT=1200
export GRASS_PNG_COMPRESSION=1
export GRASS_PNGFILE=$OUTPUT/map.png


################################################################################
###############
################################################################################
######################################################################
##########
##########  MAPPING AGRICULTURAL PRODUCTION
##########
################################################################################
######################################################################
################################################################################
###############
# Reproject administrative ward boundary layers into laea
rm -r $INPUT/WARD/ward_laea
mkdir $INPUT/WARD/ward_laea
# cd $INPUT/WARD/ward_laea
# reproject Less Favoured areas and UK ward shape file into LAEA projection
ogr2ogr     -s_srs EPSG:27700  -t_srs EPSG:3035  $INPUT/WARD/ward_laea/ward98_laea.shp
$INPUT/WARD/ward98/ward98.shp
ogr2ogr     -s_srs EPSG:27700  -t_srs EPSG:3035  $INPUT/LEAST_FAV/maglfa_laea.shp
$INPUT/LEAST_FAV/maglfa.shp
ogr2ogr     -s_srs EPSG:3035  -t_srs EPSG:3035 -select NAME  -where "NAME =
'DISADVANTAGED'"  $INPUT/LEAST_FAV/DA_laea.shp
$INPUT/LEAST_FAV/maglfa_laea.shp
ogr2ogr     -s_srs EPSG:3035  -t_srs EPSG:3035 -select NAME  -where "NAME = 'SEVERELY
DISADVANTAGED'"  $INPUT/LEAST_FAV/SDA_laea.shp
$INPUT/LEAST_FAV/maglfa_laea.shp
cd $OUTPUT

################################################################################
###########
################################################################################
###########
#  Data are now ready in LAEA projection and shp file format.
# Create a new working directory "GRASS MAPSET"
```

```
rm -r $GRASSDB/$LOCATION/$MAPSETS
g.mapset -c mapset=$MAPSETS
g.region   n=3718200 s=3095400     e=3763500   w=3203000 res=100 save=England

# inport vector file of WARDS and LEAST FAVOURED AREAS
v.in.ogr dsn=$INPUT/WARD/ward_laea/ward98_laea.shp output=ward98 -o --overwrite
v.in.ogr dsn=$INPUT/LEAST_FAV/maglfa_laea.shp output=LFarea -o --overwrite
v.in.ogr dsn=$INPUT/LEAST_FAV/SDA_laea.shp output=SDA -o --overwrite
v.in.ogr dsn=$INPUT/LEAST_FAV/DA_laea.shp output=DA -o --overwrite

# Clip corine land cover to STUDY AREA and convert ward vector map into raster
r.mapcalc clc2000_$MAPSETS = g100_00.tif@PERMANENT

# Rasterize vector ward vectors map into raster
v.to.rast input=ward98 output=wardID column=PI --overwrite
v.to.rast input=DA output=DA column=cat --overwrite
v.to.rast input=SDA output=SDA column=cat --overwrite
v.to.rast input=LFarea output=LFarea column=cat --overwrite

# CREATE MASKS according to LAND COVER TYPE CLASSIFICATION AND Less
FAVOURED AREAS (lowland, severely desadvantages, desadvantages)
r.mask -r
r.mapcalc mask_ward = "if(wardID >= 0  , 1  , null() )"
r.mask -o input=mask_ward
r.mapcalc SDA_mask = "if(SDA >= 0, 1  , null())"
r.mapcalc DA_mask = "if(DA >= 0, 1  , null())"
r.mapcalc LFarea_mask = "if(LFarea >= 0, 1  , null())"
r.mapcalc HA_PLANTATION_mask = "if(clc2000_$MAPSETS == 12, 1  , null())"
r.mapcalc HA_LIVESTOCK_mask = "if(clc2000_$MAPSETS == 18, 1  , null())"
r.mapcalc HA_COMPLEX_mask = "if(clc2000_$MAPSETS == 20, 1  , null())"
r.mapcalc HA_AGRINAT_mask = "if(clc2000_$MAPSETS == 21, 1  , null())"

for YEAR in 1990 1995 2000 2002 2005 ; do
awk -F,  ' { if(NR==4) print  "\"ID\"," $0 }'  $INPUT/AJS_ward$YEAR/
$YEAR'nuts_all_levels.csv' >  tmp1.txt
awk -F,  ' { if($6==5) print $5 "," $0 }'  $INPUT/AJS_ward$YEAR/$YEAR'nuts_all_levels.csv' |
sort -k 1,1 >> tmp1.txt
awk -F, '{gsub(/ /,"_") ; gsub(/"/,"") ; gsub(/\(/,"") ; gsub(/\)/,"") ; gsub(/\&/,"") ; gsub(/_and_/,"_") ;
gsub(/__/,"_") ; gsub(/<5/,"min_of_5") ; gsub(/</,"") ;  gsub(/#/,"NA") ; gsub(/---/,"NA") ; print}'
tmp1.txt  > head_$YEAR.csv

awk -F, '{ print  $1  "," $44 "," $46 "," $48 "," $50 "," $52 "," $56 "," $58 "," $60 "," $62 "," $64 ","
$66 "," $68 "," $70 "," $72 "," $74 "," $76 "," $78 "," $80 "," $82 "," $88 "," $90 "," $94 "," $96 ","
$98 "," $100 "," $102 "," $104 "," $106 "," $116 "," $118 "," $120 "," $124  }  ' head_$YEAR.csv  >
$OUTPUT/SYNT_$YEAR.csv
done

path=$OUTPUT
export path
R --vanilla --no-readline  -q    <<    EOF
## IMPORT DATA
mod.t = Sys.getenv(c('path'))
```

```
INPUT = as.character(mod.t[1])
H1990 = read.csv(paste(INPUT,"/SYNT_1990.csv",sep=""), header=T)
H1995 = read.csv(paste(INPUT,"/SYNT_1995.csv",sep=""), header=T)
H2000 = read.csv(paste(INPUT,"/SYNT_2000.csv",sep=""), header=T)
H2002 = read.csv(paste(INPUT,"/SYNT_2002.csv",sep=""), header=T)
H2005 = read.csv(paste(INPUT,"/SYNT_2005.csv",sep=""), header=T)

## CREATE ID UNIQUE and cumulate all datasets
ID=unique(c(H2000$ID,H2002$ID,H2005$ID))  # if mean 1990 and 1995 included, add:
H1990$ID,H1995$ID,
merge=data.frame(ID)
dat2 <- merge(dat1, H2000, all=TRUE)
dat3 <- merge(dat2, H2002, all=TRUE)
dat3 <- merge(H2000, H2002, all=TRUE)
dat <- merge(dat3, H2005, all=TRUE)

## Compute the mean ha and livestok nbr of years
mean.dat=data.frame(ID)
for (cropT in c(2:33)){ mean.dat[[cropT]]=NA
for (ward in unique(c(H2000$ID,H2002$ID,H2005$ID))) { # if average 1990 and 95 add:
H1990$ID,H1995$ID,
mean.dat[[cropT]][mean.dat$ID==ward]=mean(subset(dat[[cropT]],dat[[1]]==ward),na.rm=TRUE)
}}
names(mean.dat)=names(H2000)
mean.dat$sumPLANT=NA
mean.dat$sumANIMAL=NA
max=length(mean.dat$ID)
for (ward.id in c(1:max)){
mean.dat[ward.id,34]   = sum(mean.dat[ward.id,2:24], na.rm=TRUE)
mean.dat[ward.id,35]   = sum(mean.dat[ward.id,25:33], na.rm=TRUE)
}

## Compute the percentage ha of each crop wuithin ward  (and livestock)
pc.dat=mean.dat
for (ward.id in c(1:max)){
for (crop.t in c(2:24)){
pc.dat[ward.id,crop.t]=pc.dat[ward.id,crop.t]/pc.dat[ward.id,34]}}

for (ward.id in c(1:max)){
for (animal.t in c(25:33)){
pc.dat[ward.id,animal.t]=pc.dat[ward.id,animal.t]/pc.dat[ward.id,35]}}
pc.dat$sumPLANT=NULL
pc.dat$sumANIMAL=NULL
write.table(pc.dat,file=paste(INPUT,"/pctable.csv",sep=""),row.name=F,col.names=T,sep=",")

## Produce tables per crop type and livestock of ID and mean percentage
for (crop.t in c(2:24)){
tmp=matrix(nrow=max,ncol=4)
tmp[,1]=pc.dat[[1]]
tmp[,2]=pc.dat[[1]]
tmp[,3]=pc.dat[[crop.t]]
tmp[,4]=pc.dat[[crop.t]]
```

```
write.table(tmp,file=paste(INPUT,"/",names(pc.dat)[crop.t],sep=""),sep = ":",
na="0",row.name=F,col.name=F)
}

for (animal.t in c(25:33)){
tmp=matrix(nrow=max,ncol=4)
tmp[,1]=pc.dat[[1]]
tmp[,2]=pc.dat[[1]]
tmp[,3]=pc.dat[[animal.t]]
tmp[,4]=pc.dat[[animal.t]]
write.table(tmp,file=paste(INPUT,"/",names(pc.dat)[animal.t],sep=""), sep = ":",
na="0",row.name=F,col.name=F)
}

EOF

## write a production type list foor plants and livestock
awk -F, '{ gsub(/"/,//) ; if(NR==1) print  $44 " " $46 " " $48 " " $50 " " $52 " " $56 " " $58 " " $60 "
" $62 " " $64 " " $66 " " $68 " " $70 " " $72 " " $74 " " $76 " " $78 " " $80 " " $82 " " $88 " " $90 "
" $94 " " $96  }  ' head_$YEAR.csv > plant_list.txt
awk -F, '{ gsub(/"/,//) ; if(NR==1) print  $98 " " $100 " " $102 " " $104 " " $106 " " $116 " " $118 "
" $120 " " $124  }  ' head_$YEAR.csv > livestock_list.txt

##############################################################################
##
#################    AGRICULTURAL VALUE IN "PASTURES" LANDCOVER
#################
##############################################################################
##
### percent value of livestock
r.mask -o input=mask_ward
for type in `cat livestock_list.txt` ; do
cat $type | r.recode in=wardID out=pc_$type --overwrite
done

## Ha of pasture according to CLC
r.mask -o input=HA_LIVESTOCK_mask
r.mapcalc ward_ha_pasture = wardID
r.stats  -c -l -n fs=' ' input=ward_ha_pasture  output=tmp.txt
awk  '{ print $1 ":" $1 ":" $2 ":" $2  }' tmp.txt > recode.pasture.txt
cat recode.pasture.txt | r.recode in=wardID out=ha_pasture --overwrite

## Ha of each livestock type within CLC PASTURE class
for type in `cat livestock_list.txt` ; do
r.mapcalc ha_$type = ha_pasture*pc_$type
done

# Price of livestock per ha in lowlands
r.mask -o -i input=LFarea_mask
for type in `cat livestock_list.txt` ; do
COST=`awk -v type=$type '{ if($1==type) print $2 }' $INPUT/price_list.txt`
r.mapcalc poundsLW_$type = ha_$type*$COST
```

```
done

# Price of livestock per ha in DISADVANTAGES areas
r.mask -o  input=DA_mask
for type in `cat livestock_list.txt` ; do
COST_DA=`awk -v type=$type '{ if($1=="DA_"type) print $2 }' $INPUT/price_list.txt`
r.mapcalc poundsDA_$type = ha_$type*$COST
done

# Price of livestock per ha in SEVERELY DISADVANTAGES areas
r.mask -o  input=SDA_mask
for type in `cat livestock_list.txt` ; do
COST=`awk -v type=$type '{ if($1=="SDA_"type) print $2 }' $INPUT/price_list.txt`
r.mapcalc poundsSDA_$type = ha_$type*$COST
done

r.mask -o input=mask_ward
r.series input=`g.mlist pattern='pounds*' sep=,` output=sum_livestock method=sum  --overwrite
r.mapcalc sum_ha_pds_LIVESTOCK=sum_livestock/ha_pasture

#####################################################################################
##
#################    AGRICULTURAL VALUE IN "Non-irrigated arable land"
#################
#####################################################################################
###########
r.mask -o input=mask_ward
for type in `cat plant_list.txt` ; do
cat $type | r.recode in=wardID out=pc_$type --overwrite
done

## Ha of plants  in "Non-irrigated arable land" according to CLC
r.mask -o input=HA_PLANTATION_mask
r.mapcalc ward_ha_crops = wardID
r.stats  -c -l -n fs=' ' input=ward_ha_crops  output=tmp.txt
awk  '{ print $1 ":" $1 ":" $2 ":" $2  }' tmp.txt > recode.crops.txt
cat recode.crops.txt | r.recode in=wardID out=ha_crops --overwrite

# Pounds per ha of different productions
for type in `cat plant_list.txt` ; do
r.mapcalc ha_$type = ha_crops*pc_$type
done

for type in `cat plant_list.txt` ; do
COST=`awk -v type=$type '{ if($1==type) print $2 }' $INPUT/price_list.txt`
r.mapcalc VEGpds_$type = ha_$type*$COST
done

r.mask -o input=mask_ward
r.series input=`g.mlist pattern='VEGpds*' sep=,` output=sum_plants method=sum  --overwrite
r.mapcalc sum_ha_pds_CROPS=sum_plants/ha_crops
```

```
###################################################################################
##############
################# AGRICULTURAL VALUE IN "Complex cultivation patterns"
#################
###################################################################################
##############
# max 75% of the area 37.5% crops and 37.5% livestock

r.mask -o input=HA_COMPLEX_mask
r.mapcalc ward_ha_crops = wardID
r.stats  -c -l -n fs=' ' input=ward_ha_crops  output=tmp.txt
awk  '{ print $1 ":" $1 ":" $2*0.375 ":" $2*0.375 }' tmp.txt > recode.COMPLEXcrops.txt
cat recode.COMPLEXcrops.txt | r.recode in=wardID out=haCOMP --overwrite

# POUNDS / Ha of different productions PLANTS
for type in `cat plant_list.txt` ; do
r.mapcalc haCOMP_$type = haCOMP*pc_$type
done

for type in `cat plant_list.txt` ; do
COST=`awk -v type=$type '{ if($1==type) print $2 }' $INPUT/price_list.txt`
r.mapcalc COMPpdsV_$type = haCOMP_$type*$COST
done

# POUNDS / Ha of different productions LIVESTOCK
for type in `cat livestock_list.txt` ; do
r.mapcalc haCOMP_$type = haCOMP*pc_$type
done
for type in `cat livestock_list.txt` ; do
COST=`awk -v type=$type '{ if($1==type) print $2 }' $INPUT/price_list.txt`
r.mapcalc COMPpdsA_$type = haCOMP_$type*$COST
done

# Overall Veg + Livestock In complex
r.mask -o input=mask_ward
r.series input=`g.mlist pattern='COMPpds*' sep=,` output=sum_COMPLEX method=sum
--overwrite
r.mapcalc totCOMP_ha = haCOMP*2
r.mapcalc sum_ha_pds_COPMPLEX_ha=sum_COMPLEX/totCOMP_ha

# Only Animal in Complex
r.series input=`g.mlist pattern='COMPpdsA*' sep=,` output=sum_COMPLEX_A method=sum
--overwrite
r.mapcalc sum_ha_pds_A_COPMPLEX_ha=sum_COMPLEX_A/totCOMP_ha
# Only Veg in COMPLEX
r.series input=`g.mlist pattern='COMPpdsV*' sep=,` output=sum_COMPLEX_V method=sum
--overwrite
r.mapcalc sum_ha_pds_V_COPMPLEX_ha=sum_COMPLEX_V/totCOMP_ha

###################################################################################
##############
################# AGRICULTURAL VALUE IN "Complex cultivation patterns"
```

```
#################
####################################################################################
##############
# 25-75% of Agriculture: avg=65.5% 31.25

r.mask -o input=HA_AGRINAT_mask
r.mapcalc ward_ha_crops = wardID
r.stats  -c -l -n fs=' ' input=ward_ha_crops  output=tmp.txt
awk  '{ print $1 ":" $1 ":" $2*0.3125 ":" $2*0.3125  }' tmp.txt > recode.AGRINAT.txt
cat recode.AGRINAT.txt | r.recode in=wardID out=haAGRINAT --overwrite

# Pounds per ha of different productions
for type in `cat plant_list.txt` ; do
r.mapcalc haAGRINAT_$type = haAGRINAT*pc_$type
done

for type in `cat plant_list.txt` ; do
COST=`awk -v type=$type '{ if($1==type) print $2 }' $INPUT/price_list.txt`
r.mapcalc AGRINATpdsV_$type = haAGRINAT_$type*$COST
done


for type in `cat livestock_list.txt` ; do
r.mapcalc haAGRINAT_$type = haAGRINAT*pc_$type
done

for type in `cat livestock_list.txt` ; do
COST=`awk -v type=$type '{ if($1==type) print $2 }' $INPUT/price_list.txt`
r.mapcalc AGRINATpdsA_$type = haAGRINAT_$type*$COST
done

r.mask -o input=mask_ward
r.series input=`g.mlist pattern='AGRINAT*' sep=,` output=sum_AGRINAT method=sum
--overwrite
r.mapcalc totAGRINAT_ha = haAGRINAT*2
r.mapcalc sum_ha_pds_AGRINAT=sum_AGRINAT/totAGRINAT_ha


# Sum Veg
r.series input=`g.mlist pattern='AGRINATpdsV*' sep=,` output=sum_AGRINAT_V method=sum
--overwrite
r.mapcalc sum_ha_pds_AGRINAT_V=sum_AGRINAT_V/totAGRINAT_ha
# Sum Animal
r.series input=`g.mlist pattern='AGRINATpdsA*' sep=,` output=sum_AGRINAT_A method=sum
--overwrite
r.mapcalc sum_ha_pds_AGRINAT_A=sum_AGRINAT_A/totAGRINAT_ha


######################################################
# TOTAL AGRICULTURAL PRODUCTION VALUE £/ha
######################################################
```

```
r.series input=`g.mlist pattern='sum_ha_pds_*' sep=,` output=tmp method=sum  --overwrite
r.null map=tmp null=0
r.mask -o input=mask_ward
r.mapcalc AGRI_PROD = tmp
r.out.gdal input=AGRI_PROD output=$OUTPUT/AGRI_PROD100m.tif

r.series
input=sum_ha_pds_AGRINAT_A,sum_ha_pds_A_COPMPLEX_ha,sum_ha_pds_LIVESTOCK
output=tmp method=sum  --overwrite
r.null map=tmp null=0
r.mask -o input=mask_ward
r.mapcalc LIVESTOCK_PROD = tmp
r.out.gdal input=LIVESTOCK_PROD output=$OUTPUT/LIVESTOCK_PROD100m.tif

r.series input=sum_ha_pds_AGRINAT_V,sum_ha_pds_V_COPMPLEX_ha,sum_ha_pds_CROPS
output=tmp method=sum  --overwrite
r.null map=tmp null=0
r.mask -o input=mask_ward
r.mapcalc VEG_PROD = tmp
r.out.gdal input=VEG_PROD output=$OUTPUT/VEG_PROD100m.tif

g.region res=1000
r.mapcalc AGRI_PROD1k = AGRI_PROD
r.mapcalc VEG_PROD1k = VEG_PROD
r.mapcalc LIVESTOCK_PROD1k = LIVESTOCK_PROD
r.out.gdal input=AGRI_PROD1k output=$OUTPUT/AGRI_PROD1k.tif
r.out.gdal input=LIVESTOCK_PROD1k output=$OUTPUT/LIVESTOCK_PROD1k.tif
r.out.gdal input=VEG_PROD1k output=$OUTPUT/VEG_PROD1k.tif



################################################################################
######################################################################
##########
##########  MAPPING FLOODS MITIGATION
##########
################################################################################
######################################################################
# Download ASTER from one of the below web sites:
# http://reverb.echo.nasa.gov/reverb/
# http://gdex.cr.usgs.gov/gdex/
# http://www.jspacesystems.or.jp/ersdac/GDEM/E/index.html
# move files to ~/downloads

cd ~/downloads/
gdalwarp -s_srs EPSG:4326  -t_srs EPSG:27700   -dstnodata nan 20130126083750_1412435450.tif
ASTER27700.tif

# Create a new GRASS LOCATION with progection EPSG:27700
cp ~/downloads/ASTER27700.tif   $GRASSDB/ASTER27700.tif
cd $GRASSDB
bash   ~/scripts/create_location.sh  ASTER27700.tif   EPSG27700  $GRASSDB
```

```
LOCATION=EPSG27700
GRASSDB=$GRASSDB
echo "LOCATION_NAME: $LOCATION"                  >  $HOME/.grassrc6
echo "MAPSET: PERMANENT"                         >> $HOME/.grassrc6
echo "DIGITIZER: none"                           >> $HOME/.grassrc6
echo "GRASS_GUI: text"                           >> $HOME/.grassrc6
echo "GISDBASE: $GRASSDB"                        >> $HOME/.grassrc6
# path to GRASS binaries and libraries:
export GISBASE=/usr/lib/grass64
export PATH=$PATH:$GISBASE/bin:$GISBASE/scripts
export LD_LIBRARY_PATH="$GISBASE/lib"
export GISRC=~/.grassrc6
export GIS_LOCK=$$ # use process ID (PID) as lock file number:
export GRASS_PNGFILE=/tmp/grass6output.png # settings for graphical output to PNG file
(optional)
export GRASS_TRUECOLOR=TRUE
export GRASS_WIDTH=900
export GRASS_HEIGHT=1200
export GRASS_PNG_COMPRESSION=1
export GRASS_PNGFILE=$OUTPUT/map.png

g.mapset -c mapset=mitigation
r.mask -r
r.in.gdal -e input=ASTER27700.tif  output=aster27700 --overwrite
cd $GRASSDB/$LOCATION/mitigation/
g.region n=133504.965805  s=7025.31607002  w=131000.42271673 e=268277.21155518
res=23.94375102 align=aster27700 save=cornish_bassin --overwrite
g.remove rast=agri27700.tif
r.mapcalc CLIPDEM=aster27700
r.watershed elev=CLIPDEM drain=fdir basin=catch stream=str thresh=100 --overwrite
r.watershed elev=CLIPDEM accumulation=accumul thresh=100 --overwrite
# convert streams to vect foir visualization
r.thin in=str out=str_thin --overwrite # the stream raster usually requires thinning
r.to.vect in=str_thin out=streams feature=line --overwrite

# multipart to single part in qgis flood_warning_areas_010k.shp  ---> warn_unique_id.shp
v.in.ogr -r -o dsn=/home/ste/Documents/data/EA_UKK/warn_unique_id.shp output=warning
v.to.rast input=warning output=warning use=cat --overwrite


####### House of cornwall
# - merge all edina house tiles in one vector
# Download vector layer from http://digimap.edina.ac.uk/digimap/home and store gml files here
~/downloads/vectorMap_local/vml_41752
mkdir -p ~/downloads/vectorMap_local/tmp_out/OUT
mkdir ~/downloads/vectorMap_local/vml_41752
cd ~/downloads/vectorMap_local/vml_41752   # directory with original gml files
out=~/downloads/vectorMap_local/tmp_out
ogr2ogr     -t_srs EPSG:27700 -clipdstlayer Area -select featureDescription -where
"featureDescription = 'Building polygon'"  $out/OUT  sw97sw.gml
cd ../
cd ~/Documents/data/edina/vectorMap_local/
```

```
for fol in $(ls | grep vml) ; do
cd /home/ste/Documents/data/edina/vectorMap_local/$fol
for infile in $(ls *gml) ; do
if [ $infile != sw97sw.gml ] ; then
outfile=$(basename $infile gml)shp
ogr2ogr    -update -append -select featureDescription -where "featureDescription = 'Building
polygon'"  $out/OUT  $infile
fi
done
done
# - rasterize houses in raster format
cd ~/downloads/vectorMap_local/tmp_out/OUT/
gdal_rasterize -at -te 131000.42271673 7025.31607002 268277.21155518 133504.965805 -tr
23.94375102 23.94375102 -burn 1 -l  Area Area.shp Area.tif
r.in.gdal input=/home/ste/Documents/data/edina/vectorMap_local/tmp_out/OUT/Area.tif
output=House  --overwrite
v.in.ogr dsn=/home/ste/Documents/data/edina/vectorMap_local/tmp_out/OUT/Area.shp
output=House
##### FINISHED HOUSES

##### LANDCOVER ranking of ecosystem services for floods mitigation
# download forest data from : http://forest.jrc.ec.europa.eu/download/data/forest-data-download/
and store tile 33 in ~/downloads/CM-FMAP_2000_3500035000-AA.tif
gdalwarp -s_srs EPSG:3035  -t_srs EPSG:27700  -tr 25 25 -te 133504.5368056825
5614.2066254507517442 246504.5368056825 118614.206625450751744    ~/downloads/   CM-
FMAP_2000_3500035000-AA.tif ~/downloads/forest_25m.tif
 ~/downloads/clc06_100m.tif

# Landcover classification:
# 1 - class 1-9, 35, 37 : Urban Industrial, Road, port Airoprt, mineral extraction, Dump site,
Constrictioin site, Inland marshes, Salt marshes
# 5 - class 12, 18,20,21: Arable land, Pasture, Complex cultivation patterns.
# 7 - Class 10,11: Green urban areas, Sport and leisure facilities
# 8 - Class 21,26,27,30,37: Agri-natural, Natural grasslane, Moors and heathland, Dunes, Salt
marshes.
# 9 - Class 29: Transitional woodland-shrub
# 20 - 23,24,25: Broadleaved forest, Coniferous forest, Mixed forests
gdalwarp -s_srs EPSG:3035  -t_srs EPSG:27700  -tr 100 100 -te 133504.5368056825
5614.2066254507517442 246504.5368056825 118614.206625450751744
~/downloads/g100_06.tif
 ~/downloads/clc06_100m.tif

r.in.gdal  input=~/downloads/clc06_100m.tif output=clc1  --overwrite
r.mapcalc clc2 = "if( clc1 < 9.0 , 1, clc1)"
r.mapcalc clc3 = " if( clc2 == 10 || clc2 == 11, 7, clc2 )"
r.mapcalc clc4 = "if( clc3 >= 12 && clc3 <= 20 , 5 , clc3 )"
r.mapcalc clc5 = "if(clc4 >= 23 && clc4 <= 25, 20, clc4 )"
r.mapcalc clc6 = "if(clc5 == 29, 9, clc5 )"
r.mapcalc clc7 = "if(clc6 >= 35 && clc6 != 37, 1, clc6 )"
r.mapcalc clc8 = "if(clc7 == 21 || clc7 == 26 || clc7 == 27 || clc7 == 30 || clc7 == 37 , 8, clc7)"
r.in.gdal  input=~/downloads/forest_25m.tif output=forest25  --overwrite
r.mapcalc CLC_weight = "if(forest25  <= 12 , 20, clc8) "
```

```
##### Lancover FINISHED

## Slope 2 classes > or < 10%
r.slope.aspect elevation=CLIPDEM slope=r_slope --overwrite
r.mapcalc slope_weight = "if(r_slope <= 10 , 1, 2) "

##### Water accumulation weight
r.mapcalc 'log_accumulation=log(abs(accumul)+1)'
r.mapcalc 'inf_rivers=if(log_accumulation>6)' # This is rivers
r.mapcalc accumul_NOweight = "if(inf_rivers != 1 , accumul, 1) "
r.mapcalc accumul_weight = "if(accumul_NOweight <= 1, 1, if(accumul_NOweight <= 3,2,3)) "


echo Waterbasin,HOUSEDENSITY > house_density.txt
for i in $(r.category map=warning | awk '{print $1}') ; do
r.mask -o input=warning maskcats=$i
HOUSEDENSITY=$(r.sum rast=House | awk '{gsub(/.000000/,"") ; print $3}')
echo $i,$HOUSEDENSITY >> house_density.txt

if [ $HOUSEDENSITY -eq 0 ] ; then
r.mask -r
r.mapcalc mit1_$i = 0
else
r.mapcalc warning_house_$i = "if(int(House) > 0 , 1 , 0)"
r.mask -o input=warning_house_$i maskcats=1
r.mapcalc 'tmpAccumul = accumul'
r.mask -r
max=$(r.info map=tmpAccumul  | grep max | awk '{print $10}')
r.mapcalc xAccumul = "if( tmpAccumul == $max ,1, 0 )"
r.out.xyz  input=xAccumul  | grep "|1" > tmp_coor.txt
EASTcoor=$(awk -F"|" '{print $1}' tmp_coor.txt)
NORTcoor=$(awk -F"|" '{print $2}' tmp_coor.txt)
r.water.outlet drain=fdir east=$EASTcoor north=$NORTcoor basin=basin$i  --overwrite
r.mask -o input=basin$i
r.mapcalc mit1_$i = "int(if(CLC_weight == 1.0 , 0.0 ,
CLC_weight*accumul_weight*slope_weight))"
r.mapcalc mit_P1_$i = "int(accumul_weight*slope_weight)"
fi
done
r.mask -r

r.mapcalc cum_temp = 0
r.mapcalc cum_P_temp = 0
for i in $(r.category map=warning | awk '{print $1}') ; do
# for i in $(seq 1 10) ; do
VALUE=$(grep -v  Category house_density.txt | awk -v i=$i -F"," '{ if($1==i)  print $2 }' |  head -1)
if [ $VALUE  -gt 0 ] ; then
echo BASSIN  $i - VAL $VALUE
r.null map=mit1_$i null=0
r.mapcalc cum_$i="cum_temp+(mit1_$i*$VALUE)"
g.remove rast=cum_temp
g.copy rast=cum_$i,cum_temp
```

```
r.null map=mit_P1_$i null=0
r.mapcalc cum_P_$i="cum_temp+(mit1_$i*$VALUE)"
g.remove rast=cum_P_temp
g.copy rast=cum_P_$i,cum_P_temp

fi
done
g.copy rast=cum_temp,A_MITIGATION
g.copy rast=cum_P_temp,P_MITIGATION

g.region res=1000
r.mapcalc A_MITIGATION_1k=A_MITIGATION
r.mapcalc P_MITIGATION_1k=P_MITIGATION

r.out.gdal  input=A_MITIGATION output=$OUTPUT/mitigation_1k.tif
r.out.gdal  input=P_MITIGATION output=$OUTPUT/mitigation_P_1k.tif

g.region res=23.94375102
r.out.gdal  input=cum_temp output=$OUTPUT/mitigation_24m.tif
r.out.gdal  input=cum_P_temp output=$OUTPUT/mitigation_P_24m.tif


####################################################################################
##########################################################################
##########
##########  MAPPING PLANT PRODUCTION (sum of positive NDVI)
##########
####################################################################################
##########################################################################
# Download data
#This script downloads Modis tiles covering the CORNWALL
# Originally Written by Hans Bosch and Sonya Ahamed for AfSIS project, Columbia University ,
modified by S. Casalegno to compute accumulate posotive NDVI values
#Project contact: Markus Walsh, Columbia University
#Pre condition:
#    Adequate space needs to be allocated for downloads and temp files
#    Write permissions to file system
#    The variable tileList needs to contain the exact name of the tile
#    Network access to Modis ftp server ftp://e4ftl01.cr.usgs.gov
#    Requires curl to be installed see http://curl.haxx.se
#
#Post condition:
#    Base directory is created with multiple date sub directories
#    Sub directories created and populated with desired tiles
#    Script builds directories starting from script execution point
#
#    Select the zone of interest from this file http://gis.cri.fmach.it/data/modis_sinusoidal.zip
#    Declare tiles to download in variable tileList
#
#    your working dir is named after modis product name: (see
https://lpdaac.usgs.gov/products/modis_products_table)
```

```
#    Create root directory for downloads
https://lpdaac.usgs.gov/products/modis_products_table/mod13q1
#
mkdir ~/download/MODIS/
cd ~/download/MODIS/
tileList=(h18v03 h17v03 h17v4) # My zone of interest
baseDir=MOD13Q1.005            # equal to MODIS product name
mkdir $baseDir
cd $baseDir
#Determine the directory dates for $baseDir
curl ftp://e4ftl01.cr.usgs.gov/MOLT/$baseDir/ --user anonymous:user@mailservice.com > dirDates
for eachDate in `cat dirDates|awk '{print $8}'` ; do #Process each date in the $baseDir
echo $eachDate
mkdir $eachDate
cd $eachDate    # Now that we have the dates get the directory listing under that date
curl ftp://e4ftl01.cr.usgs.gov/MOLT/$baseDir/$eachDate/ --user anonymous:user@mailservice.com
> allFiles4Date
grep hdf allFiles4Date > files2Get         # From all the files only get the *.hdf and *.hdf.xml files
   for eachTile in ${tileList[*]} ; do     # Get only the tiles needed
   grep $eachTile files2Get > tiles2Get
     for eachFile in `cat tiles2Get|awk '{print $8}'` ; do
     curl -O ftp://e4ftl01.cr.usgs.gov/MOLT/$baseDir/$eachDate/$eachFile --user
anonymous:user@mailservice.com
     done
   done
cd ..  #Change directory up one to base dir
done

# create a template file with all pixels = 0 values
eachDate=2000.02.18
gdal_merge.py -o ../../MOD13Q1.005_TIF/template_modis_proj.tif $(ls
../../MOD13Q1.005_TIF/NDVI/*tif)
gdalwarp -s_srs '+proj=sinu +R=6371007.181 +nadgrids=@null +wktext' -t_srs EPSG:27700 -r
cubic  ../../MOD13Q1.005_TIF/template_modis_proj.tif
../../MOD13Q1.005_TIF/template_27700.tif
g.mapset -c mapset=NDVI
r.in.gdal -e input=~/downloads/MODIS/MOD13Q1.005_TIF/template_27700.tif output=template
g.region rast=template  nsres=236.13777419 ewres=236.13777419
r.mapcalc template = "0"

for eachDate in `cat dirDates|awk '{print $8}'` ; do #Process each date in the $baseDir
echo $eachDate
cd $eachDate
for file in *.hdf ; do
gdal_translate  'HDF4_EOS:EOS_GRID:"'$file'":MODIS_Grid_16DAY_250m_500m_VI:250m 16
days NDVI' ../../MOD13Q1.005_TIF/NDVI/NDVI_$eachDate$(basename $file .hdf)".tif"
done
gdal_merge.py -o ../../MOD13Q1.005_TIF/NDVI_Merge/NDVI$eachDate.tif $(ls
../../MOD13Q1.005_TIF/NDVI/*tif)
gdalwarp -s_srs '+proj=sinu +R=6371007.181 +nadgrids=@null +wktext' -t_srs EPSG:27700 -r
cubic   ../../MOD13Q1.005_TIF/NDVI_Merge/NDVI$eachDate.tif
../../MOD13Q1.005_TIF/NDVI_Merge/NDVI_import.tif
```

```
r.external input=/disk2/MODIS/MOD13Q1.005_TIF/NDVI_Merge/NDVI_import.tif output=import
--overwrite
r.mapcalc out = "if(  import >= 0 , template+import  , template )"
r.mapcalc template = out
rm /disk2/MODIS/MOD13Q1.005_TIF/NDVI_Merge/*
rm /disk2/MODIS/MOD13Q1.005_TIF/NDVI/*
cd ../
done

r.out.gdal input=template output=$OUTPUT/NDVI_sum.tif

##################################################################################
########################################################################
##########
##########  HARMONIZE ALL DATA to process them in zonation
##########
##################################################################################
########################################################################

mkdir ~/harmonized
cd ~/harmonized
WKDIR=~/harmonized
# AGRICULTURE
gdalwarp -s_srs EPSG:3035  -t_srs EPSG:27700  -tr 1000 1000 -te 133504.5368056825
5614.2066254507517442 246504.5368056825 118614.206625450751744  -dstnodata nan
$OUTPUT/AGRI_PROD_tot.tif agri27700.tif

# RECREATION see Casalegno S, Inger R, DeSilvey C, Gaston KJ (2013) Spatial Covariance
between Aesthetic Value & Other Ecosystem Services. PLoS One 8: e68437.
gdalwarp -r cubic  -s_srs EPSG:4326 -t_srs EPSG:27700    -dstnodata 101
input/UPLOADER_UK_1km.tif  recr27700.tif

# SOIL download http://eusoils.jrc.ec.europa.eu/library/esdac/index.html
# reference: Jones RJA, Hiederer R, Rusco E, Loveland PJ, Montanarella L (2005) Estimating
organic carbon inthe soils of Europe for policy support. Eur J Soil Sci 56: 655–671 and store here
$OUTPUT/soil_carbon_jrc.tif
gdalwarp  -s_srs EPSG:3035  -t_srs EPSG:27700  -tr 1000 1000 -te 133504.5368056825
5614.2066254507517442 246504.5368056825 118614.206625450751744  -dstnodata nan
$OUTPUT/soil_carbon_jrc.tif soil27700.tif

# SOLAR download here
http://re.jrc.ec.europa.eu/pvgis/download/solar_radiation_classic_laea_download.html and store in
$OUTPUT/G_opt_classic_latlon_year.asc
gdalwarp -s_srs EPSG:4326 -t_srs EPSG:27700  -tr 1000 1000 -te 133504.5368056825
5614.2066254507517442 246504.5368056825 118614.206625450751744  -dstnodata nan
$OUTPUT/G_opt_classic_latlon_year.asc sola27700.tif

# MASK download administrative boundary from here
https://geoportal.statistics.gov.uk/geoportal/catalog/main/home.page stroe it here
$INPDIR/cornwall_admin27700.shp
gdal_rasterize  -te 133504.5368056799925398 5614.2066254499950446
246504.5368056799925398 118614.2066254499950446 -tr 1000 1000  -burn 1  -l
```

```
cornwall_admin27700 $INPDIR/cornwall_admin27700.shp   admin27700.tif

# CORINE LAND COVER 100M RES
gdalwarp -s_srs EPSG:3035 -t_srs EPSG:27700  -tr 100 100 -te 133504.5368056825
5614.2066254507517442 246504.5368056825 118614.206625450751744  -dstnodata nan
$INPDIR/g100_06.tif CLC_100m_27700.tif

# new working environment
g.mapset -c mapset=zonation

# inport solar irradiation,  agricultural value, soil carbon
# create a mask and mask it
for SERVICE in sola agri recr soil ; do
r.in.gdal  input=$INPDIR/$SERVICE"27700.tif" output=$SERVICE  --overwrite
done
r.in.gdal  input=$INPDIR/admin27700.tif output=admin  --overwrite
r.buffer -z input=admin output=adminBuff distances=1000
r.mapcalc mask_admin = "if( adminBuff >= 0 , 1, null() )"
r.mask -o input=mask_admin
r.mapcalc NEW=recr*1.0
g.remove rast=recr
g.rename rast=NEW,recr


#######################################################################################
##########################################
#######################################################################################
##########################################
# recode maps 1-100
# export asc files
for SERVICE in sola agri recr soil ; do
max=$(r.info $SERVICE | grep max | awk '{print $10}')
min=$(r.info $SERVICE | grep max | awk '{print $7}')
r.mapcalc $SERVICE"_M1"="(($SERVICE-$min)/($max-$min))*100"
rm $WKDIR/$SERVICE.asc
rm $WKDIR/$SERVICE.asc.aux.xml
rm $WKDIR/$SERVICE.prj
r.out.gdal  input=$SERVICE"_M1" format=AAIGrid type=Float32 output=$WKDIR/
$SERVICE.asc
done

# convert no data from "-9999" to "-nan"
for SERVICE in sola agri recr soil wind ; do
awk '{gsub(/-nan/,"-9999") ; print$0'} $WKDIR/$SERVICE.asc >  $WKDIR/$SERVICE"_m9.asc"
done
cd $WKDIR/output
rm $(ls | grep -v m9)
cd $WKDIR
mv  $WKDIR/sola_m9.asc  $WKDIR/potential_solar.asc
mv  $WKDIR/wind_m9.asc  $WKDIR/potential_wind.asc
mv  $WKDIR/soil_m9.asc  $WKDIR/carbon_soil.asc
mv  $WKDIR/recr_m9.asc  $WKDIR/culture_aesthetic.asc
```

```
mv  $WKDIR/agri_m9.asc  $WKDIR/agriculture.asc
rm soil.* agri.* recr.* wind.* sola.*


################################################################################
######################################################################
##########
##########  MAPPING Wind energy production (other environmental service)
##########
################################################################################
######################################################################
# Download pdf  http://www.cornwall.gov.uk/media/6851869/wind-turbine-operational.pdf
# rasterize locations of wind turbines save csv file here $INPDIR/turbine_MW.csv
r.in.xyz --overwrite input=$INPDIR/turbine_MW.csv output=turbine z=4 method=sum fs=,
r.sum rast=turbine
SERVICE=turbine
max=$(r.info $SERVICE | grep max | awk '{print $10}')
min=0.0001
r.recode input=$SERVICE output=recod_$SERVICE --overwrite << EOF
 $min:$max:0.0:100.0
EOF
r.null map=recod_$SERVICE null=0
rm $WKDIR/recod_$SERVICE.asc
r.out.gdal  input=recod_$SERVICE format=AAIGrid type=Float32
output=$WKDIR/recod_$SERVICE.asc
awk '{gsub(/-nan/,"-9999") ; print$0'} $WKDIR/recod_$SERVICE.asc >
$WKDIR/realized_wind.asc
rm $WKDIR/recod_$SERVICE.*


################################################################################
##################################################################
##########
########## MAP Solar energy production (other environmental service)
##########
################################################################################
##################################################################
# Download locations from here http://www.cornwall.gov.uk/media/6011289/PV-sites-pre-apps-
pub-available.pdf
# and digitize locations ad save it $INPDIR/PV_panels27700.shp
v.in.ogr dsn=$INPDIR/PV_panels27700.shp output=PV --overwrite
v.db.addcol PV col="area_ha double precision"
v.to.db map=PV type=centroid option=area col=area_ha unit=h
v.out.ascii input=PV fs=, dp=3 columns=area_ha > $WKDIR/pv_ha.csv
r.in.xyz --overwrite input=$WKDIR/pv_ha.csv output=PVha z=4 method=sum fs=,
rm $WKDIR/pv_ha.csv
r.mapcalc realized_photovoltaic = PVha*sola
SERVICE=realized_photovoltaic
max=$(r.info $SERVICE | grep max | awk '{print $10}') #= 14.612   18089.36

# max=14.613
min=0.000
```

```
r.recode input=$SERVICE output=recod_$SERVICE --overwrite << EOF
 $min:$max:0.0:100.0
EOF
r.null map=recod_$SERVICE null=0

r.out.gdal  input=recod_$SERVICE format=AAIGrid type=Float32 output=$WKDIR/tmpPV.asc
awk '{gsub(/-nan/,"-9999") ; print$0'} $WKDIR/tmpPV.asc >  $WKDIR/realized_photovoltaic.asc
rm $WKDIR/tmpPV.*

################################################################################
###########################
################################################################################
###########################
##########
########## MAPPING RECREATION (cultural service)
##########
################################################################################
###########################
################################################################################
###########################
## Digitize the following layers
# - National trust gardens  http://www.nationaltrust.org.uk/visit/places/gardens-and-parks/
# - English Heritage register of parks and garden (http://www.geostore.com/environment-agency
# - Golf courts  ttp://www.cornwall-online.co.uk/golf/
# and save vector files here ~/shapes
mkdir ~/shapes
for file in $(ls *.kmz) ; do
cp $file shapes/$(basename $file kmz)zip
cd shapes
unzip $(basename $file kmz)zip
mv doc.kml $(basename $file kmz)kml
rm $(basename $file kmz)zip
ogr2ogr  $(basename $file kmz)shp $(basename $file kmz)kml
cd ..
done
rm shapes/*kml
cd shapes
mkdir merged
ls *.shp > list_shp.txt
ogr2ogr -s_srs EPSG:4326 -t_srs EPSG:27700 Nat_trust_gardens.shp $(cat list_shp.txt | head -1)
for infile in $(cat list_shp.txt) ; do
if [ $infile != $(cat list_shp.txt | head -1) ] ; then
echo $infile
ogr2ogr -s_srs EPSG:4326 -t_srs EPSG:27700 -update -append   Nat_trust_gardens.shp $infile
fi
rm $(echo $(basename $infile .shp).*)
done
mv Nat_trust_gardens.* ../
rm -r shapes
cd /home/ste/Documents/data/EA_UKK/recreation
ls *.shp > list
for infile in $(cat list) ; do
```

```
rm $(echo $(basename $infile .shp)_cw.*)
ogr2ogr -t_srs EPSG:27700 -clipsrc ~/shapes/cornwall_admin27700.shp $(echo $(basename
$infile .shp)_cw.shp)  $infile
done

ogr2ogr -t_srs EPSG:27700 -update -append   sport_leisure.shp Nat_trust_gardens.shp
ogr2ogr -t_srs EPSG:27700 -update -append   sport_leisure.shp  golf27700.shp
ogr2ogr -t_srs EPSG:27700 -update -append   sport_leisure.shp  regd_parks_gardens_cw.shp


SERVICE=culture_recreation
v.in.ogr  dsn=$INPDIR/sport_leisure.shp output=$SERVICE  --overwrite
v.mkgrid map=gridnew grid=113,113
v.overlay ainput=gridnew atype=area alayer=1 binput=$SERVICE btype=area blayer=1
output=intersect operator=and --overwrite
v.db.addcol intersect col="SURFACE double precision"
v.to.db map=intersect type=centroid option=area col=SURFACE unit=h
v.out.ascii input=intersect fs=, dp=3 columns=SURFACE > $WKDIR/$SERVICE"_ha.csv"
r.in.xyz --overwrite input=$WKDIR/$SERVICE"_ha.csv" output=$SERVICE z=4 method=sum
fs=,
rm $WKDIR/$SERVICE"_ha.csv"
max=$(r.info $SERVICE | grep max | awk '{print $10}')
min=$(r.info $SERVICE | grep max | awk '{print $7}')
r.recode input=$SERVICE output=recod_$SERVICE --overwrite << EOF
 $min:$max:0.0:100.0
EOF
r.out.gdal  input=recod_$SERVICE format=AAIGrid type=Float32 output=$WKDIR/tmp.asc
awk '{gsub(/-nan/,"-9999") ; print$0'} $WKDIR/tmp.asc >  $WKDIR/$SERVICE.asc
rm $WKDIR/tmp.*


##########################################################################################
############################
##########################################################################################
############################
#####
##### Map tourism (cultural service)
#####
##########################################################################################
############################
##########################################################################################
############################
## DOWNLOAD MENE DATABASE sav format from here
http://www.naturalengland.org.uk/ourwork/evidence/mene.aspx and save data in ~/MENE/.
R --vanilla --no-readline   -q  << EOF
library(foreign)
xx=read.spss("~/MENE//121002_visit_based.sav")
xx1=read.spss("~/MENE//121002_VISIT_forLA.sav")
xx1=read.spss("~/MENE//RES.sav")
X=xx1[[191]]
Y=xx1[[192]]
region=xx1[193]
```

```
county=xx1[194]
year=xx1[3]
regionwhere=xx1[47]
origin=xx1[175]   # $ District2009ofresidence
Q7=xx1[48] # Q7 County where the visit was taken
MILES=xx1[49] # Q8 distance in miles
MENE=matrix(NA,nrow=160376,ncol=3)
MENE[,1]=X
MENE[,2]=Y
MENE[,3]=xx1[[49]] # distance
write.table(MENE,file="~/MENE/miles.csv",row.names=F,col.names=F)
EOF

awk '{if($1!=0) print $0}' miles.csv  > proces/XYdist.txt
r.in.xyz --overwrite input=$(awk '{if($1!=0) print $0}' ~/MENE/miles.csv) output=tourism z=3
method=n fs=space
SERVICE=culture_visitors
v.in.ogr -r dsn=$INPDIR/distance.shp output=$SERVICE --overwrite
v.out.ascii input=$SERVICE fs=, dp=3 columns=x > $WKDIR/$SERVICE.csv
r.in.xyz --overwrite input=$WKDIR/$SERVICE.csv output=$SERVICE z=4 method=n fs=,
rm $WKDIR/$SERVICE.csv
max=$(r.info $SERVICE | grep max | awk '{print $10}')
min=1
r.recode input=$SERVICE output=recod_$SERVICE --overwrite << EOF
 $min:$max:1.0:100.0
EOF
r.null map=recod_$SERVICE null=0
r.out.gdal  input=recod_$SERVICE format=AAIGrid type=Float32
output=$WKDIR/tmp$SERVICE.asc
awk '{gsub(/-nan/,"-9999") ; print$0'} $WKDIR/tmp$SERVICE.asc >  $WKDIR/
$SERVICE"_m9.asc"
rm $WKDIR/tmp$SERVICE.*


######################################################################################
#############################
######################################################################################
#############################
#####
##### Map Aboveground carbon (regulating service)
#####
######################################################################################
#############################
######################################################################################
#############################
g.mapset -c mapset=biomass
r.mask -o input=mask_admin@zone
g.region res=100
r.in.gdal  input=$INPDIR/CLC_100m_27700.tif output=clc1  --overwrite
g.region res=25
r.mapcalc biom_grass_1 = "if( clc1 >= 10.0 && clc1 <= 14.0 || clc1 >= 18.0 && clc1 <= 22.0 || clc1
== 26, 0.0625  , 0.0)"  # 1t/ 100m2 (ha) = 0.0625t / 25m2
```

```
r.mapcalc biom_shrub_2 = "if( clc1 >= 15.0 && clc1 <= 16.0 || clc1 == 27 || clc1 == 29 || clc1 ==
32, 0.125  , 0.0)" # 2t/ 100m2 (ha) = 0.125t / 25m2
r.mapcalc biom_bro_56 = "if( clc1 == 23.0 , 3.5  , 0.0)" # 56t/ 100m2 (ha) = 3.5 t / 25m2
r.mapcalc biom_con_24 = "if( clc1 == 24.0 , 1.5  , 0.0)" # 24t/ 100m2 (ha) = 1.5 t / 25m2
r.series input=biom_grass_1,biom_shrub_2,biom_bro_56,biom_con_24     output=Other_biom
method=sum --overwrite
gdalwarp -s_srs EPSG:3035  -t_srs EPSG:27700  -tr 25 25 -te 133504.5368056825
5614.2066254507517442 246504.5368056825 118614.206625450751744   $INPDIR/CM-
FTYP_2006_3500035000.tif $INPDIR/forest_25m.tif
r.in.gdal  input=$INPDIR/forest_25m.tif output=forest25  --overwrite
r.mapcalc biomass_25 = "if( forest25 == 11.0 , 3.5  , if( forest25 == 12.0 , 1.5  , Other_biom ) )"
g.region res=100
r.resamp.stats input=biomass_25 output=biomass_100 method=sum --overwrite
g.region res=1000
r.resamp.stats input=biomass_25 output=biomass_1km method=sum --overwrite
SERVICE=carbon_vegetation
max=$(r.info $SERVICE | grep max | awk '{print $10}')
min=0.001
r.recode input=$SERVICE output=recod_$SERVICE --overwrite << EOF
 $min:$max:1.0:100.0
EOF
r.null map=recod_$SERVICE null=0
r.out.gdal  input=recod_$SERVICE format=AAIGrid type=Float32
output=$WKDIR/tmp$SERVICE.asc
awk '{gsub(/-nan/,"-9999") ; print$0'} $WKDIR/tmp$SERVICE.asc >
$WKDIR/carbon_vegetation.asc
rm $WKDIR/tmp*

##################################################################################
###########################
##################################################################################
###########################
#####
##### Map urban development (other environmental service)
#####
##################################################################################
###########################
##################################################################################
###########################
g.mapset -c mapset=urban
r.mask -o input=mask_admin@zone
g.region res=100
g.copy rast=clc1@biomass,clc100m
r.mapcalc urbR_100m = "if(clc100m <= 6.0 || clc100m >= 9.0 && clc100m <= 11.0 , 1  , 0.0)"
g.region res=1000
r.resamp.stats input=urbR_100m output=urbR method=sum --overwrite
r.out.gdal  input=urbR format=AAIGrid type=Float32 output=$WKDIR/tmpR.asc
awk '{gsub(/-nan/,"-9999") ; print$0'} $WKDIR/tmpR.asc  >  $WKDIR/realized_urban.asc
r.mapcalc urbPtemp=100-urbR
r.mapcalc urbP="if(urbPtemp == 100.0 , 0 ,urbPtemp )"
r.out.gdal  input=urbP format=AAIGrid type=Float32 output=$WKDIR/tmpP.asc
awk '{gsub(/-nan/,"-9999") ; print$0'} $WKDIR/tmpP.asc >  $WKDIR/potential_urban.asc
```

```
rm $WKDIR/tmp*

##############################################################################
# Harmonize FLOOD MITIGATION
##############################################################################
r.mask -o input=mask_admin@zone
r.in.gdal input=$INPDIR/mitigation_1k.tif output=realized_mitigation --overwrite
r.in.gdal input=$INPDIR/mitigation_P_1k.tif output=potential_mitigation --overwrite

for SERVICE in realized_mitigation potential_mitigation ; do
max=$(r.info $SERVICE | grep max | awk '{print $10}')
min=0.001
r.recode input=$SERVICE output=recod_$SERVICE --overwrite << EOF
 $min:$max:1.0:100.0
EOF
r.null map=recod_$SERVICE null=0
r.out.gdal  input=recod_$SERVICE format=AAIGrid type=Float32
output=$WKDIR/tmp$SERVICE.asc
awk '{gsub(/-nan/,"-9999") ; print$0'} $WKDIR/tmp$SERVICE.asc >  $WKDIR/$SERVICE.asc
g.remove rast=recod_$SERVICE
done
rm $WKDIR/tmp*

##############################################################################
# Harmoniize Plant productivity
##############################################################################
r.mask -o input=mask_admin
res=1000
r.in.gdal input=$OUTPUT/NDVI_sum.tif output=ndvi --overwrite
SERVICE=plant_production
r.mapcalc $SERVICE = ndvi
max=$(r.info $SERVICE | grep max | awk '{print $10}')
min=0.001
r.recode input=$SERVICE output=recod_$SERVICE --overwrite << EOF
 $min:$max:1.0:100.0
EOF
r.null map=recod_$SERVICE null=0
r.out.gdal  input=recod_$SERVICE format=AAIGrid type=Float32
output=$WKDIR/tmp$SERVICE.asc
awk '{gsub(/-nan/,"-9999") ; print$0'} $WKDIR/tmp$SERVICE.asc >  $WKDIR/$SERVICE.asc
rm $WKDIR/tmp*
g.remove rast=recod_$SERVICE

### CREATE zones of cornwall
r.in.gdal input=~/download/coast_27700_1k.tif output=coast
idval=2
for file in west lizard central east_north east_south  ; do
name=$file
v.in.ogr dsn=$WKDIR/input/CORN_AREAS/$name.shp output=$name --overwrite
v.to.rast input=$name output=ZONE_$name use=val value=$idval --overwrite
idval=$((idval+1))
done
```

```
r.patch input=ZONE_central,ZONE_east_north,ZONE_east_south,ZONE_lizard,ZONE_west
out=ZONES5 --overwrite
r.mapcalc Zt = "if( ZONES5 == 3 , 2, if( ZONES5 == 4,3, if( ZONES5 == 5 || ZONES5 == 6 ,4,
ZONES5 )))"
r.mapcalc ZONES = "if( coast == 0 , Zt, coast )"
SERVICE=ZONES
g.remove
rast=ZONE_central,coast,ZONE_lizard,ZONE_west,ZONE_east_north,ZONE_east_south,ZONES
5,Zt
r.out.gdal  input=ZONES format=AAIGrid type=Float32 output=$WKDIR/tmp$SERVICE.asc
awk '{gsub(/-nan/,"-9999") ; print$0'} $WKDIR/tmp$SERVICE.asc >  $WKDIR/ZONES"_m9.asc"
rm $WKDIR/tmp$SERVICE.asc

##### Run zonation using different weights and PRINT output maps from zonation
g.mapset -c mapset=print_review
OUTDIR=~/out_reviewpaper
mkdir -p ~/out_reviewpaper

echo 0 0:0:0 > $OUTDIR/zone_palet.txt  # black
echo 0.199999 0:0:0 >> $OUTDIR/zone_palet.txt  # black
echo 0.2 0:0:127 >> $OUTDIR/zone_palet.txt  # dark blue
echo 0.499999 0:0:127 >> $OUTDIR/zone_palet.txt  # dark blue
echo 0.5 0:0:255 >> $OUTDIR/zone_palet.txt  # light blue
echo 0.749999 0:0:255 >> $OUTDIR/zone_palet.txt  # light blue
echo 0.75 255:255:0 >> $OUTDIR/zone_palet.txt  # yellow
echo 0.899999 255:255:0 >> $OUTDIR/zone_palet.txt  # yellow
echo 0.90 255:0:255 >> $OUTDIR/zone_palet.txt  # magenta
echo 0.949999 255:0:255 >> $OUTDIR/zone_palet.txt  # magenta
echo 0.95 153:0:0 >> $OUTDIR/zone_palet.txt  # brown
echo 0.979999 153:0:0 >> $OUTDIR/zone_palet.txt  # brown
echo 0.98 255:0:0 >> $OUTDIR/zone_palet.txt  # red
echo 1.0 255:0:0   >> $OUTDIR/zone_palet.txt  # red
echo nv 255:255:255   >> $OUTDIR/zone_palet.txt  # red

INP=/zonation/
cd $INP
for i in R_agri_W_2.CAZ_E.prop R_agri_Wneg_2.CAZ_E.prop R_carb_W_5.CAZ_E.prop
R_carb_Wneg_5.CAZ_E.prop R_cult_W_10.CAZ_E.prop R_cult_Wneg_2.CAZ_E.prop
R_mitR_W_10.CAZ_E.prop R_mitR_Wneg_5.CAZ_E.prop R_prod_W_10.CAZ_E.prop
R_prod_Wneg_5.CAZ_E.prop R_rene_W_10.CAZ_E.prop R_rene_Wneg_5.CAZ_E.prop
R_urbR_W_10.CAZ_E.prop R_urbR_Wneg_2.CAZ_E.prop R_noweight.CAZ_E.prop ; do

title=$(echo $i | awk '{gsub(/R/,""); gsub(/Wneg_/," -"); gsub(/W_/," +"); gsub(/.CAZ_E.prop/,"");
gsub(/_agri_/,"Agriculture"); gsub(/_carb_/,"Carbon stocks"); gsub(/_cult_/,"Cultural serv.");
gsub(/_mit_/,"Flood mitigation"); gsub(/_prod_/,"Plant production"); gsub(/_rene_/,"Renewable
energy") ; gsub(/_urb_/,"Urban development"); ; gsub(/_noweight/,"Unweighted"); print }')
r.in.gdal input=$i.asc output=$i -o --overwrite
cat $OUTDIR/zone_palet.txt | r.colors map=$i color=rules
d.mon start=png1
d.rast $i bg=255:255:255
d.text -b -p text="$title" size=6 color=black bgcolor=white  at=50,40
d.mon stop=png1
```

```
mv  map.png $OUTDIR/png_maps/$i.png
echo $out
done

cd  /home/ste/EcoServ/zonation/out_reviewpaper/png_maps/
cp ../../out_3/png_maps/palette.png .
ls   *png > ../list_png.txt
# ls   Ra* > ../list_RaP4.txt

montage -geometry 640x480 $(cat ../list_png.txt) zonation15maps.png
montage -geometry 640x480 R_agri_W_2.CAZ_E.prop.png R_agri_Wneg_2.CAZ_E.prop.png
R_carb_W_5.CAZ_E.prop.png R_carb_Wneg_5.CAZ_E.prop.png
R_mitR_W_10.CAZ_E.prop.png R_mitR_Wneg_5.CAZ_E.prop.png
R_prod_W_10.CAZ_E.prop.png R_prod_Wneg_5.CAZ_E.prop.png
R_cult_W_10.CAZ_E.prop.png R_cult_Wneg_2.CAZ_E.prop.png R_rene_W_10.CAZ_E.prop.png
R_rene_Wneg_5.CAZ_E.prop.png R_urbR_W_10.CAZ_E.prop.png
R_urbR_Wneg_2.CAZ_E.prop.png R_noweight.CAZ_E.prop.png palette.png
Figure4_zonemap.png
```

Data processing scripting routine (R codes)
http://www.r-project.org/

```
### Data processing for Regional scale prioritization for key ecosystem services, renewable energy
production and urban development
# # Before running this script you need to compile maps following the MAPPING.sh script to
download data and process maps

###################### Inport data compute rho p-val n of spatial covariance
library(rgdal) #import data
library(raster) # create raster stack and Moran I
library(SpatialPack) # compute crh stats
## import environmental service layers
map_names=c("agri_m9.asc","carA_m9.asc","carS_m9.asc","mitR_m9.asc","prod_m9.asc","pvl_R
_m9.asc","recA_m9.asc","recL_m9.asc","recT_m9.asc","urbR_m9.asc","winR_m9.asc","ZONES_
m9.asc")

## FIGURE 1 - Plot environmental services and zones of Cornwall
import12=vector("list",12)
for ( x in c(1:12) ){
import12[[x]]=raster(readGDAL(paste("~/harmonized/",map_names[x],sep="")))
}
MAPS=brick(import12[[1]],import12[[2]],import12[[3]],import12[[4]],import12[[5]],import12[[6]]
,import12[[7]],import12[[8]],import12[[9]],import12[[10]],import12[[11]],import12[[12]])
import12[[5]]@data@values[which(complete.cases(import12[[1]]@data@values)==FALSE)]=NA
mycolor=topo.colors(100)
mycolor[1]="#808080" # grey
TITLE=c("Carbon in soil: 0.72","Agriculture: 0.58","Urban development: 0.47","Carbon above
ground: 0.47","Plant production: 0.41","Aesthetic: 0.38","Recreation: 0.36","Flood mitigation:
0.26","Tourism: 0.18","Wind energy: 0.16","Solar energy: 0.11","Zones of Cornwall")
nn=0
png(paste("~/figures/Figure1_maps.png",sep=""), width = 1600, height = 1200)
nf <- layout(matrix(c(1:12),3,4,byrow=TRUE), c(1,1), c(1,1), TRUE) ; layout.show(nf) ;
par(mar=c(5,5,3,5))
for ( x in c(3,1,10,2,5,7,8,4,9,11,6) ){
nn=1+nn
r=import12[[x]]
plot(r, col=mycolor, legend=F, axes=F, main=TITLE[nn],cex.main=3, cex=2, cex.legend=3, box=F)
if (x==6){plot(r, legend.only=TRUE, col=mycolor, legend.width=6, legend.shrink=0.75,
axis.args=list(at=c(0,50,89),  labels=c(0,50,100),  cex.axis=2.2))}
}
r=import12[[12]]
plot(r, col=c("blue","red","green3","orange"), legend=F, axes=F, main="Zones of
Cornwall",cex.main=3, cex=1.8, box=F)
legend(131000,110000,c("Coastal","West","Centre","East"), bty = "n",  pch=c(15,15,15,15),
pt.cex=c(2.5,2.5,2.5),  cex=3,  col=c("blue","red","green3","orange"))
dev.off()

#######################################################################################
##########
##### Compute Moran I index
MI=matrix(ncol=1,nrow=11)
```

```
rownames(MI)=c("Agriculture value","Carbon above ground","Carbon in soil","Flood mitigation","Plant production","Solar energy","Aesthetic","Recreation","Tourism","Urban development","Wind energy")
for ( x in c(1:12) ){
MI[x,1]=Moran(import12[[x]])
}
MI[order(MI[,1]),]
# 6         Solar energy 0.1075170
# 11         Wind energy 0.1595617
# 9              Tourism 0.1822565
# 4     Flood mitigation 0.2570684
# 8           Recreation 0.3586911
# 7            Aesthetic 0.3799540
# 5     Plant production 0.4123475
# 2  Carbon above ground 0.4679798
# 10   Urban development 0.4710979
# 1          Agriculture 0.5768623
# 3       Carbon in soil 0.7203855
# 12   Zones of Cornwall 0.7508789


##############################################################################################
#### FIGURE 2 - Boxplot of maps, zonal statistics
TITLE=c("Agriculture value","Carbon above ground","Carbon in soil","Flood mitigation","Plant production","Solar energy","Aesthetic","Recreation","Tourism","Urban development","Wind energy","Zones of Cornwall")

png("~/figures/Figure2_stat.png", width = 800, height = 900)
nf <- layout(matrix(c(1:12),4,3,byrow=TRUE), c(1,1), c(1,1), TRUE) ; layout.show(nf) ; par(mar=c(5,5,3,1))
for (band in c(1,2,3,4,5,7,8,9,10,11,6)) {
COS=subset(na.omit(MAPS@data@values[,band][MAPS@data@values[,12]==1]), na.omit(MAPS@data@values[,band][MAPS@data@values[,12]==1]>0))
WES=subset(na.omit(MAPS@data@values[,band][MAPS@data@values[,12]==2]), na.omit(MAPS@data@values[,band][MAPS@data@values[,12]==2]>0))
CEN=subset(na.omit(MAPS@data@values[,band][MAPS@data@values[,12]==3]), na.omit(MAPS@data@values[,band][MAPS@data@values[,12]==3]>0))
EAS=subset(na.omit(MAPS@data@values[,band][MAPS@data@values[,12]==4]), na.omit(MAPS@data@values[,band][MAPS@data@values[,12]==4]>0))
ALL=subset(na.omit(MAPS@data@values[,band][MAPS@data@values[,12]==4]), na.omit(MAPS@data@values[,band][MAPS@data@values[,12]==4]>0))
boxplot(COS,WES,CEN,EAS,ALL, col=(c("blue","red","green4","orange","grey")),main=TITLE[band], cex.main=2,cex.axis=1.5)
}
plot(1,1,type="n",xlab="",ylab="",axes=F)
legend(0.7,1.5,c("Coastal","West","Centre","East","Overall"), bty = "n",  pch=c(15,15,15,15), pt.cex=c(2.5,2.5,2.5),  cex=2.5,  col=c("blue","red","green4","orange","gray"))
dev.off()


####################################################################################
```

```
##########
#### FIGURE 3 - Plot percentils value of service vs other services
IN.path=("~/inputs")
OUT.path=("~/figures")
qtl0100=read.table(paste(IN.path,"/Bsum_0100Pquant.csv",sep=""), header = TRUE, sep =",")
layers=unique(qtl0100$Other_Eco_serv)
names_ES=c("Carbon above ground","Carbon in soil","Flood mitigation","Plant production","Solar energy","Aestetic","Recreation","Tourism","Urban development","Wind energy","Agriculture")
colore=c("green2","chocolate4","chocolate1","blue","green4","yellow","gray","gray10","gray40","red","skyblue")
pchtype=c(17,15,15,15,23,17,17,17,15,21,21)
leg=c("Agriculture","Carbon above ground","Carbon in soil","Flood mitigation","Plant production","solar energy","Aestetic","Recreation","Tourism","Urban development","Wind energy")
xLABEL=c("Agriculture value","Carbon above ground value","Carbon in soil value","Floods mitigation value","Plant production value","Aestetic value","Recreation value","Tourism value","Urban development value","Wind energy value","Solar energy")
yLABEL=c("co-occurring service % value","","","co-occurring service % value","","","co-occurring service % value","","","co-occurring service % value","","")

png(paste(OUT.path,"/Figure3_cooc.png",sep=""), width = 1200, height = 1600)
nf <- layout(matrix(c(1:12),4,3,byrow=TRUE), c(1,1), c(1,1), TRUE) ; layout.show(nf) ;
par(mar=c(7,6,1,1))
cnt=0
for (Mlay in c(11,1,2,3,4,6,7,8,9,10,5) ){
cnt=cnt+1
PLO=subset(qtl0100,qtl0100$Rank_layer==layers[Mlay])
if (cnt!=11)
{y.axis.vect=c(0,round(max(PLO$Other_percent)/3),round(max(PLO$Other_percent)/3)*2,round(max(PLO$Other_percent)))}
else{y.axis.vect=c(0,round(max(PLO$Other_percent)/3,2),round(max(PLO$Other_percent)/3,2)*2,round(max(PLO$Other_percent),2))}
plot(PLO$quantils,PLO$Other_percent,type="n",xlab=xLABEL[cnt],
ylab=yLABEL[cnt],cex.main=3,cex.axis=1.2,cex.lab=2.9,xaxt="n",yaxt="n",font.lab=2)
axis(1,at=c(1,25,50,75,99),labels=c("min","","","","max"),cex.axis=2.5)
axis(2,at=y.axis.vect,labels=y.axis.vect,cex.axis=2.5)
for(i in 1:nlevels(PLO$Other_Eco_serv)){
points(PLO$quantils[PLO$Other_Eco_serv==levels(PLO$Other_Eco_serv)[i]],PLO$Other_percent[PLO$Other_Eco_serv==levels(PLO$Other_Eco_serv)[i]],col=colore[i],pch=pchtype[i], type="o")
abline(v=75,lty=2)
}
}
plot(c(1:5),c(1:5), type="n", axes=F, xlab="",ylab="")
legend(1,5.4,leg, bty = "n",
col=c("green2","chocolate4","chocolate1","blue","green4","yellow","gray","gray10","gray40","red","skyblue"), pch=c(17,15,15,15,19,17,17,17,15,19,19), pt.cex=c(3,3,3,3,3,3,3,3,3,3),
pt.lwd=c(2,2,2,2,2,2,2,2,2,2), cex=3)
dev.off()

##########################################################################################
```

```
## FIGURE 6 zonation courves according to different weights
# After running zonation, pre-process inputs in bash terminal using the following commands
#
#OUTPATH=~/input/sens_v3
#cd $OUTPATH
#mkdir figures
#mkdir csv_table
# for i in $(ls R*curves*) ; do
# out=$(echo $(basename $i .CAZ_E.curves.txt).csv)
# awk '{if(NR>=2) print $1 ","
$2","$3","$4","$5","$6","$7","$8","$9","$10","$11","$12","$13","$14","$15","$16","$17","$18","$
19}' $i > $out
# done

# 1 - Prop_landscape_lost
# 2 - cost_needed_for_top_fraction
# 3-  min_prop_rem
# 4 - ave_prop_rem
# 5 - W_prop_rem
# 6 - ext-1
# 7 - ext-2
# prop for each species remaining at level of removal
#for i in $(ls *curves*) ; do
#out=$(echo $(basename $i .CAZ_E.curves.txt).csv)
#awk '{if(NR>=2) {for (i=1; i <= NF-1; i++) printf $i","; print NL }}' $i > csv_table/$out
#echo $i
#done

#cd csv_table
#ls A* > listA.txt
#ls P* > listP.txt
#ls R* > listR.txt


### load weighted inputs
TABLE.path=as.character("~/input/csv_table/")
name_tab=c("Prop_land","cost","min_prop_rem","ave_prop_rem","W_prop_rem","ext1","ext2","A
gri","carA","carS","mitR","prod","pvl_R","recA","recL","recT","urbR","winR")
R_names=c("agri_W_","agri_Wn","carb_W_","carb_Wn","cult_W_","cult_Wn","mitR_W_","mitR
_Wn","prod_W_","prod_Wn","rene_W_","rene_Wn","urbR_W_","urbR_Wn")
Rlist=read.table(paste(TABLE.path,"listR.txt",sep=""))
REALIZED=c(as.character(Rlist$V1))
tab_R <- vector("list", length(REALIZED))
names(tab_R)=REALIZED
noW=grep("R_noweight.csv",REALIZED)
NWG=read.table(paste(TABLE.path,REALIZED[noW],sep="")  , head=F, skip=0 ,sep="," )
names(NWG)=name_tab
syntCUM <- vector("list",14)
syntWEI <- vector("list",14)

for (g14 in R_names ){ sim_id=grep(g14,REALIZED);
if (g14 == "agri_W_" || g14 == "agri_Wn" ) {w.layer="Agri"}
```

```
if (g14 == "carb_W_" || g14 == "carb_Wn" ) {w.layer=c("carS","carA")}
if (g14 == "mitR_W_" || g14 == "mitR_Wn" ) {w.layer="mitR"}
if (g14 == "prod_W_" || g14 == "prod_Wn" ) {w.layer="prod"}
if (g14 == "rene_W_" || g14 == "rene_Wn" ) {w.layer=c("pvl_R","winR")}
if (g14 == "urbR_W_" || g14 == "urbR_Wn" ) {w.layer="urbR"}
if (g14 == "cult_W_" || g14 == "cult_Wn" ) {w.layer=c("recA","recL","recT")}

syntCUM[[which(R_names==g14)]]=c(1:13)
if (length(w.layer)==1) { syntCUM[[which(R_names==g14)]][1]=sum(NWG[8:18])-
sum(NWG[[w.layer]]) }
if (length(w.layer)==2) { syntCUM[[which(R_names==g14)]][1]=sum(NWG[8:18])-
sum(NWG[[w.layer[1]]]+NWG[[w.layer[2]]]) }
if (length(w.layer)==3) { syntCUM[[which(R_names==g14)]][1]=sum(NWG[8:18])-
sum(NWG[[w.layer[1]]]+NWG[[w.layer[2]]]+NWG[[w.layer[3]]]) }
syntWEI[[which(R_names==g14)]]=c(1:13)
if (length(w.layer)==1){ syntWEI[[which(R_names==g14)]][1]=sum(NWG[[w.layer]]) }
if (length(w.layer)==2){ syntWEI[[which(R_names==g14)]][1]=sum(NWG[[w.layer[1]]]
+NWG[[w.layer[2]]]) }
if (length(w.layer)==3){ syntWEI[[which(R_names==g14)]][1]=sum(NWG[[w.layer[1]]]
+NWG[[w.layer[2]]]+NWG[[w.layer[3]]]) }

ix=0
NV=c(13,4,5,2,6,7,8,3,9,10,11,12)
for (sim12 in sim_id ){
ix=ix+1
tab_R[[sim12]]=read.table(paste(TABLE.path,REALIZED[sim12],sep="")  , head=F, skip=0
,sep="," )
names(tab_R[[sim12]])=name_tab
if (length(w.layer)==1){ syntCUM[[which(R_names==g14)]][NV[ix]]=sum(tab_R[[sim12]]
[,8:18])-sum(tab_R[[sim12]][,w.layer]) }
if (length(w.layer)==2){ syntCUM[[which(R_names==g14)]][NV[ix]]=sum(tab_R[[sim12]]
[,8:18])-sum(tab_R[[sim12]][,w.layer[1]]+tab_R[[sim12]][,w.layer[2]]) }
if (length(w.layer)==3){ syntCUM[[which(R_names==g14)]][NV[ix]]=sum(tab_R[[sim12]]
[,8:18])-sum(tab_R[[sim12]][,w.layer[1]]+tab_R[[sim12]][,w.layer[2]]+tab_R[[sim12]]
[,w.layer[3]]) }

if (length(w.layer)==1){ syntWEI[[which(R_names==g14)]][NV[ix]]=sum(tab_R[[sim12]]
[,w.layer])}
if (length(w.layer)==2){ syntWEI[[which(R_names==g14)]][NV[ix]]=sum(tab_R[[sim12]]
[,w.layer[1]]+tab_R[[sim12]][,w.layer[2]]) }
if (length(w.layer)==3){ syntWEI[[which(R_names==g14)]][NV[ix]]=sum(tab_R[[sim12]]
[,w.layer[1]]+tab_R[[sim12]][,w.layer[2]]+tab_R[[sim12]][,w.layer[3]]) }
}
}

leg=c("Agriculture","Carbon above ground","Carbon in soil","Floods mitigation","Plant
production","Solar energy","Aestetic","Leisure","Tourism","Urban development","Wind energy")
XLABEL=c("","","","","","","","","","","","","Proportion of Landscape removed","Proportion of
Landscape removed","Proportion of Landscape removed","Proportion of Landscape removed")
YLABEL=c("Service value remaining","","","","Service value remaining","","","","Service value
remaining","","","","Service value remaining","","","")
```

```
png("/home/../figures/Fig_6_zonation_weights.png", width = 1200, height = 1200)
count=0
nf <- layout(matrix(c(1:16),4,4,byrow=TRUE), c(1,1), c(1,1), TRUE) ; layout.show(nf) ;
par(mar=c(5,5,3,1))
#
map2plot=c("R_carb_W_5.csv","R_carb_Wneg_2.csv","R_cult_W_5.csv","R_mitR_W_40.csv","R_prod_W_5.csv","R_rene_W_5.csv","R_urbR_W_40.csv","R_urbR_Wneg_2.csv")
map2plot=c("R_agri_W_2.csv","R_agri_Wneg_2.csv","R_carb_W_5.csv","R_carb_Wneg_5.csv","R_mitR_W_10.csv","R_mitR_Wneg_5.csv","R_prod_W_10.csv","R_prod_Wneg_5.csv","R_cult_W_10.csv","R_cult_Wneg_2.csv","R_rene_W_10.csv","R_rene_Wneg_5.csv","R_urbR_W_10.csv","R_urbR_Wneg_2.csv")
TITLE=c("Agriculture +2","Agriculture -2","Carbon +5","Carbon -5","Flood mitigation +10","Flood mitigation -5","Plant production +10","Plant production -5","Culture +10","Culture -2","Renewable energy +10","Renewable energy -5","Urban development +10","Urban development -2")
for (map in map2plot ){
count=count+1
ilt=gsub(".csv", "",map)
# par(mar=c(5,5,5,1))
plot(tab_R[[map]][[1]],tab_R[[map]][[8]], pch=20,col="green3", main=TITLE[count], cex.main=2, xlab=XLABEL[count] , ylab=YLABEL[count] , cex.lab=2,cex.axis=1.3) #agri
points(tab_R[[map]][[1]],tab_R[[map]][[9]],col="chocolate4",pch=20,cex=1) #carbon Aerial
points(tab_R[[map]][[1]],tab_R[[map]][[10]],col="chocolate1",pch=21,cex=1) #carbon Soil
points(tab_R[[map]][[1]],tab_R[[map]][[11]],col="blue",pch=3,cex=1) # mitigation
points(tab_R[[map]][[1]],tab_R[[map]][[12]],col="green4",pch=20,cex=1) # production
points(tab_R[[map]][[1]],tab_R[[map]][[13]],col="yellow",pch=20,cex=1) #  photovoltaic
points(tab_R[[map]][[1]],tab_R[[map]][[14]],col="gray",pch=3,cex=1) #  Aestetic
points(tab_R[[map]][[1]],tab_R[[map]][[15]],col="gray20",pch=3,cex=0.8) #  Leisure
points(tab_R[[map]][[1]],tab_R[[map]][[16]],col="gray50",pch=21,cex=1.5) #  Tourism
points(tab_R[[map]][[1]],tab_R[[map]][[17]],col="red",pch=19,cex=1) #  Urban
points(tab_R[[map]][[1]],tab_R[[map]][[18]],col="skyblue",pch=21,cex=1.5) #  Wind
}
tab=NWG
plot(tab[[1]],tab[[8]], pch=20,col="green3",main="Unweighted", xlab="Proportion of Landscape removed" , ylab="", cex.lab=2,cex.axis=1.3,cex.main=2) #agri
points(tab[[1]],tab[[9]],col="chocolate4",pch=20,cex=1) #carbon Aerial
points(tab[[1]],tab[[10]],col="chocolate1",pch=21, cex=1) # carbon soil
points(tab[[1]],tab[[11]],col="blue",pch=3, cex=1) # mitigation
points(tab[[1]],tab[[12]],col="green4",pch=20,cex=1) # production
points(tab[[1]],tab[[13]],col="yellow",pch=20,cex=1) # photovoltaic
points(tab[[1]],tab[[14]],col="gray",pch=3,cex=1) # Aestetic
points(tab[[1]],tab[[15]],col="gray20",pch=3,cex=0.8)# Leisure
points(tab[[1]],tab[[16]],col="gray50",pch=21,cex=1.5) # Tourism
points(tab[[1]],tab[[17]],col="red",pch=19,cex=1) # Urban
points(tab[[1]],tab[[18]],col="skyblue",pch=21,cex=1.5)


plot(c(1:5),c(1:5), type="n",axes=F,xlab="",ylab="")
legend(1,5.2,leg, bty = "n",
col=c("green3","chocolate4","chocolate1","blue","green4","yellow","gray","gray20","gray50","red","skyblue"), pch=c(19,19,19,19,19,19,19,19,19,19,19),
pt.cex=c(2.5,2.5,2.5,2.5,2.5,2.5,2.5,2.5,2.5,2.5), pt.lwd=c(2,2,2,2,2,2,2,2,2,2), cex=2)
```

```
dev.off()


#### POLOT cum value vs weights per each weight type ANNEX FIGURE S"
leg.txt=c("Not weighted Ecosystem Services","Weighted ecosystem services")
leg=c("Agriculture","Agriculture negative","Carbon","Carbon negative","Cultural","Cultural negative","Flood mitigation","Flood mitigation negative","Plant production","Plant producton neg.","Renewable energy","Renewable energy negative","Urban development","Urban development neg.")

png("~/figures/weight_treshold_annex.png", width = 800, height = 1200)
# postscript("/home/ste/EcoServ/PAPERS/Cornwall_ecoserv_zonation/figures/weight_treshold_annex2.ps", width = 2400, height = 1600)
treshold=c(1,1,2,2,3,1,3,2,3,2,3,2,3,1)
nbr=0
nf <- layout(matrix(c(1:15),5,3,byrow=TRUE), c(1,1), c(1,1), TRUE) ; layout.show(nf) ;
par(mar=c(5,5,3,1))
for (g14 in R_names ){
nbr=nbr+1
i2p=which(R_names==g14)
max.y=max(c(syntWEI[[i2p]]*10,syntCUM[[i2p]]))+100
min.y=min(c(syntWEI[[i2p]]*10,syntCUM[[i2p]]))-100
# png(paste(OUTpath,g14,"_sensV3.png",sep=""), width = 800, height = 800)

plot(c(1:13),syntWEI[[i2p]]*10,col="red",type="l",main=leg[i2p],ylim=c(min.y,max.y),ylab="Cumulate value",xlab="Weight",axes=F)
points(c(1:13),syntCUM[[which(R_names==g14)]],type="l")
axis(1,at=c(1,2,3,4,5,7,9,11),label=c(2,5,10,20,30,50,70,90),cex.axis=1)
axis(2) #,at=c(6000,6500,7000,7500,7800),label=c("min","","","","max"),cex.axis=1)
abline(v=treshold[nbr],col=4,lty=2,lwd=3)
box()
}
plot(c(1:5),c(1:5), type="n",ylim=c(1,5))
legend(1,5,c("Other Ecosystem Services","Weighted Eco. Services"), bty = "n",
col=c("black","red"), pch=c(19,19), pt.cex=c(2,2), pt.lwd=c(2,2), cex=1.5)
dev.off()

### plot only floods mitigation
png("~/figures/Figure5_sens.png", width = 800, height = 800)
par(mar=c(5,5,1,1))
g14="mitR_W_"
i2p=which(R_names==g14)
max.y=max(c(syntWEI[[i2p]]*10,syntCUM[[i2p]]))+100
min.y=min(c(syntWEI[[i2p]]*10,syntCUM[[i2p]]))-100
plot(c(1:13),syntWEI[[i2p]]*10,col="red",type="l",ylim=c(min.y,max.y),ylab="Cumulate service value",xlab="Weight factor", axes=F, cex.lab=2,cex.main=2, cex=2,lwd=5)
points(c(1:13),syntCUM[[which(R_names==g14)]],type="l",lwd=5)
axis(1,at=c(1,2,3,4,5,7,9,11),label=c(2,5,10,20,30,50,70,90),cex.axis=2)
axis(2,at=c(6000,6500,7000,7500,7800),label=c("min","","","","max"),cex.axis=2)
abline(v=3,col=4,lty=2,lwd=3)
```

```
legend(5,7900,c("Other co-occurring services","Flood mitigation"), bty = "n", col=c("black","red"),
pch=c(19,19), pt.cex=c(2,2), pt.lwd=c(2,2), cex=2.5)
box()
dev.off()




##############################################################################
#############
##### Compute modifieed correlation CRH test on ranked data and coorect for multiple tests
##############################################################################
#############

zMAP=vector("list",5)
zMAP[[5]]=matrix(ncol=12,nrow=12769)
zCORR=vector("list",5)
zPVAL=vector("list",5)
coords=matrix(ncol=2,nrow=12769)
coords=xyFromCell(MAPS,1:12769)

for ( ZONE in c(1:4)){
zMAP[[ZONE]]=MAPS[MAPS@data@values[,12]==ZONE]
zCORR[[ZONE]]=matrix(ncol=11,nrow=11)
zPVAL[[ZONE]]=matrix(ncol=11,nrow=11)
for ( x in c(1:11)){
for ( y in c(1:11)){
xdata=na.omit(data.frame(zMAP[[ZONE]][,x],data.frame(zMAP[[ZONE]]
[,y],coords[which(MAPS@data@values[,12]==ZONE),1],coords[which(MAPS@data@values[,12]
==ZONE),2])))
temp=modified.ttest(rank(xdata[[1]]),rank(xdata[[2]]),matrix(ncol=2,nrow=length(xdata[[1]]),c(xda
ta[[3]],xdata[[4]])))
zCORR[[ZONE]][x,y]=temp$corr
zPVAL[[ZONE]][x,y]=temp$p.value
}}}

ZONE=5
zMAP[[ZONE]]=MAPS@data@values
zCORR[[ZONE]]=matrix(ncol=11,nrow=11)
zPVAL[[ZONE]]=matrix(ncol=11,nrow=11)
for ( x in c(1:11)){for ( y in c(1:11)){
xdata=na.omit(data.frame(zMAP[[ZONE]][,x],data.frame(zMAP[[ZONE]]
[,y],coords[,1],coords[,2])))
temp=modified.ttest(rank(xdata[[1]]),rank(xdata[[2]]),matrix(ncol=2,nrow=length(xdata[[1]]),c(xda
ta[[3]],xdata[[4]])))
zCORR[[ZONE]][x,y]=temp$corr
zPVAL[[ZONE]][x,y]=temp$p.value
}}
save(zCORR,file="/home/ste/EcoServ/PAPERS/Cornwall_ecoserv_zonation/TABLES/correlation_
data")
save(zPVAL,file="/home/ste/EcoServ/PAPERS/Cornwall_ecoserv_zonation/TABLES/pvalue_data"
)
```

```
Pvalue=data.frame(c(1:55),c(1:55),c(1:55),c(1:55),c(1:55))
for (ZONE in seq(1,5)) {
Pvalue[[ZONE]]=zPVAL[[ZONE]][lower.tri(zPVAL[[ZONE]]==TRUE)]
}

colnames(Pvalue)= c("Coast","West","Central","East","Overall")
P_03=Pvalue
P_03[P_03<0.001]=3
P_03[P_03<0.01]=2
P_03[P_03<0.05]=1
P_03[P_03<1]=0

# See Benjamini Y, Hochberg Y (1995) Controlling the false discovery rate: a practical and
powerful approach to multiple testing. J Roy Statist Soc Ser B 57: 289–300.
# for details on the p value correction for multiple tests
FDRvalue=Pvalue
for (x in 1:55){
FDRvalue[x,]=p.adjust(FDRvalue[x,],method="fdr",n=5)
}
FDR_03=FDRvalue
FDR_03[FDR_03<0.001]=3
FDR_03[FDR_03<0.01]=2
FDR_03[FDR_03<0.05]=1
FDR_03[FDR_03<1]=0

# Count significant not significant correlations
SigP_03=P_03
SigFDR_03=FDR_03
SigFDR_03[FDR_03>0]=1
SigP_03[SigP_03>0]=1

SigFDR_03[[6]]=0
SigP_03[[6]]=0
P_03[[6]]=0
for (x in 1:55){
SigP_03[[6]][x]=sum(SigP_03[x,])
P_03[[6]][x]=sum(P_03[x,])
SigFDR_03[[6]][x]=sum(SigFDR_03[x,])
}

# Number of  significant correlations
sum(SigP_03[,1])
sum(SigP_03[,2])
sum(SigP_03[,3])
sum(SigP_03[,4])
sum(SigP_03[,5])
sum(SigP_03[,6])

# Number of significant correlations p-value
length(P_03[[6]][P_03[[6]]>0])
length(FDR_03[[6]][FDR_03[[6]]>0])
```

```
length(P_03[[6]][P_03[[6]]==0])
length(FDR_03[[6]][FDR_03[[6]]==0])

### Stationarity summary
statio=data.frame(c(1:55),c(1:55),c(1:55),c(1:55),c(1:55))
colnames(statio)= c("Coast","West","Central","East","Overall")
for (ZONE in seq(1,5)) {
statio[[ZONE]]=zCORR[[ZONE]][lower.tri(zCORR[[ZONE]]==TRUE)]
}

average.val=c(1:55) ## average and st dev ( between all)
sd.val=c(1:55) ## average and st dev ( between all)
avg=statio
for (x in 1:55){
avg[x,][which(is.na(statio[x,])==T)]=100
if(sum(avg[x,])==500){
average.val[x]=NA
sd.val[x]=NA
}
else
average.val[x]=mean(as.numeric(avg[x,which(avg[x,]!=100)][1:5]))
sd.val[x]=sd(as.numeric(avg[x,which(avg[x,]!=100)][1:5]))
}


for (x in 1:5){  ## remove not significant correlations
statio[[x]][which(FDR_03[[x]]==0)]=NA
statio[[x]][which(P_03[[x]]==0)]=NA
}

maxval=c(1:55) ## max correlation value ( between significant)
sta=statio
for (x in 1:55){
sta[x,][which(is.na(statio[x,])==T)]=100
if(sum(sta[x,])==500)
maxval[x]=NA
else
maxval[x]=sta[x,which(abs(sta[x,])==max(abs(sta[x,which(sta[x,]!=100)])))]
}

maxzone=as.character(c(1:55)) ## zone where we found max correlation
sta=statio
for (x in 1:55){
sta[x,][which(is.na(statio[x,])==T)]=100
if(sum(sta[x,])==500)
maxzone[x]="NA"
else
maxzone[x]=names(sta[which(abs(sta[x,])==max(abs(sta[x,which(sta[x,]!=100)])))])
}

plevel=as.character(c(1:55)) ### significance level of max correlationm
sta=statio
```

```
for (x in 1:55){
sta[x,][which(is.na(statio[x,])==T)]=100
if(sum(sta[x,])==500)
plevel[x]="NA"
else
plevel[x]=P_03[x,which(abs(sta[x,])==max(abs(sta[x,which(sta[x,]!=100)])))]
}

maxCorr=matrix(ncol=11,nrow=11)
nb_sig_zone=matrix(ncol=11,nrow=11)  ## nbr of significant zones per correlation
nb_sig_zone[lower.tri(maxCorr)==TRUE] = SigP_03[,6]
nb_sig_zone=t(nb_sig_zone)

p_levelCorr=matrix(ncol=11,nrow=11) # p-value significance of the maximum correlations
p_levelCorr[lower.tri(maxCorr)==TRUE] = plevel
p_levelCorr=t(p_levelCorr)

zoneCorr=matrix(ncol=11,nrow=11) # Zone where the correlation was highest
zoneCorr[lower.tri(maxCorr)==TRUE] = maxzone
zoneCorr=t(zoneCorr)

maxCorr=matrix(ncol=11,nrow=11) # correlation value
maxCorr[lower.tri(maxCorr)==TRUE] = maxval

avg.matrix=matrix(ncol=11,nrow=11)
avg.matrix[lower.tri(avg.matrix)==TRUE] = average.val

sd.matrix=matrix(ncol=11,nrow=11)
sd.matrix[lower.tri(sd.matrix)==TRUE] = sd.val


## PRINT TABLE IN LATEX format
colnames(maxCorr)=c("Agri","Car A","Car
S","Miti","Prod","Sol","Aest","Recr","Turism","Urban","Wind")
rownames(maxCorr)=c("Agri","Car A","Car
S","Miti","Prod","Sol","Aest","Recr","Turism","Urban","Wind")
colnames(nb_sig_zone)=c("Agri","Car A","Car
S","Miti","Prod","Sol","Aest","Recr","Turism","Urban","Wind")
rownames(nb_sig_zone)=c("Agri","Car A","Car
S","Miti","Prod","Sol","Aest","Recr","Turism","Urban","Wind")
colnames(zoneCorr)=c("Agri","Car A","Car
S","Miti","Prod","Sol","Aest","Recr","Turism","Urban","Wind")
rownames(zoneCorr)=c("Agri","Car A","Car
S","Miti","Prod","Sol","Aest","Recr","Turism","Urban","Wind")
colnames(p_levelCorr)=c("Agri","Car A","Car
S","Miti","Prod","Sol","Aest","Recr","Turism","Urban","Wind")
rownames(p_levelCorr)=c("Agri","Car A","Car
S","Miti","Prod","Sol","Aest","Recr","Turism","Urban","Wind")
colnames(sd.matrix)=c("Agri","Car A","Car
S","Miti","Prod","Sol","Aest","Recr","Turism","Urban","Wind")
rownames(sd.matrix)=c("Agri","Car A","Car
S","Miti","Prod","Sol","Aest","Recr","Turism","Urban","Wind")
```

```
colnames(avg.matrix)=c("Agri","Car A","Car
S","Miti","Prod","Sol","Aest","Recr","Turism","Urban","Wind")
rownames(avg.matrix)=c("Agri","Car A","Car
S","Miti","Prod","Sol","Aest","Recr","Turism","Urban","Wind")
write.table(format(maxCorr, digits=1),file="~/TABLES/MAXcorr.txt",sep=",")
write.table(zoneCorr,file="~/TABLES/ZONEcorr.txt",sep=",")
write.table(format(p_levelCorr, digits=1),file="~/TABLES/Plevel_corr.txt",sep=",")
write.table(nb_sig_zone,file="~/TABLES/NBR_significant_corr.txt",sep=",")
write.table(avg.matrix,file="~/TABLES/avg_corr.txt",sep=",")
write.table(sd.matrix,file="~/TABLES/sd_corr.txt",sep=",")
```
